# A niche that triggers aggressiveness within BRCA1-IRIS overexpressing triple negative tumors is supported by reciprocal interactions with the microenvironment

**DOI:** 10.18632/oncotarget.20892

**Published:** 2017-09-14

**Authors:** Daniel Ryan, Danielle Bogan, Joanna Davies, Jim Koziol, Wael M. ElShamy

**Affiliations:** ^1^ Breast Cancer Program, San Diego Biomedical Research Institute, San Diego, CA, USA; ^2^ University of Mississippi Medical Center, Jackson, MS, USA; ^3^ Department of Molecular and Experimental Medicine, Scripps Research Institute, San Diego, CA, USA

**Keywords:** BRCA1-IRIS, macrophages, mesenchymal stem cells, metastasis, Il-1beta

## Abstract

Production of metastasis capable precursors begins within the primary tumor. Here, we define the bidirectional interactions with stromal cells involved in promoting these precursors within BRCA1-IRIS (hereafter IRIS) overexpressing (IRISOE) TNBC tumors. We define an aggressiveness niche, functionally defined as the necrotic/hypoxic core of the tumor, in which metabolically stressed, hypoxic, and inflamed IRISOE TNBC cells secrete higher levels of cytokines, chemokines and growth factors. One cytokine; IL-1β attracts mesenchymal stem cells (MSCs) to the niche and activates them to secrete CXCL1 that entrains IRISOE cells to secrete higher levels of CCL2 and VEGF. CCL2 attracts macrophages (TAMs) to the niche and activates them to secrete S100A8, and VEGF attracts endothelial cells (ECs) and activates them to secrete IL-8. In concert, CXCL1, S100A8 and IL-8 entrain aggressiveness in IRISOE TNBC cells within the niche. Indeed, compared to IRISOE cells alone, tumors developed by co-injecting IRISOE cells admixed with MSCs (10:1) in athymic mice were bigger and more aggressive. They contained more TAMs and ECs, expressed higher-levels of basal, epithelial to mesenchymal transition, and stemness biomarkers, quickly progressed to lymph-node or visceral metastases, and were highly sensitive to the IL-1β inhibitor “Anakinra”. Our findings supported by human data show that breast cancer patients with high-levels of IL-1β, CXCL1, CCL2, S100A8, VEGF, and IL-8 would show worse clinical outcomes. Our findings argue that this cytokine set is a diagnostic biomarker for patients who may benefit from an IRIS inhibitor-based therapy, and is a blue print for translation of approaches to combining that therapy with inhibitors of these bidirectional interactions to overcome TNBC metastasis.

## INTRODUCTION

While intrinsic abilities to grow, and disseminate are possessed by breast tumor cells, extrinsic abilities imposed by their bidirectional interactions with the surrounding stromal cells; e.g., carcinoma associated fibroblasts (CAFs), mesenchymal stem cells (MSCs), and tumor associated macrophages (TAM) also exacerbate aggressiveness in these tumor cells [1-5].

MSCs, which was isolated from bone marrow (BM), adipose tissue, among other tissues [6] are capable of self-renewal and differentiation into several cell types, e.g., adipocytes, osteocytes, and fibrocytes [4, 7]. MSCs under pathological conditions, such as tissue injury or cancer, are mobilized towards the site of damage attracted by the pro-inflammatory environment [8], such as increased local or systemic interleukin-1 beta (IL-1β). This primary inflammation driver signals through IL-1R to promote a variety of cellular functions [9, 10], e.g., activation of MSCs in aggressive breast cancers [3, 11-13]. MSCs activated by IL-1β secrete other inflammatory cytokines, such as CXCL1 [14-16], which is implicated through signaling through CXCR2 expressed on breast cancer cells in the dissemination, poor patient prognosis, chemo-resistance, and metastasis [14, 17]. Therapeutic targeting of the CXCL1/CXCR2 circuit in an adjuvant setting circumvents chemotherapy resistance in breast cancer patients [14, 17].

Tumor-induced immune dysfunction is a serious challenge in cancer immunotherapy [18]. Tumor-associated macrophages (TAMs) is a key player in promoting this immune dysfunction leading to enhanced breast cancer aggressiveness [18]. Monocytes chemoattractant protein (MCP1/CCL2) is a key chemokine regulating monocytes infiltration into tumors [19, 20]. CCL2 is secreted by a variety of immune, stromal, and malignant cells leading to recruitment of TAMs to sites of chronic inflammation within breast tumors [21-23] to promote progression and metastasis [23]. Interestingly, while luminal A/ER^+^-tumors support macrophages anti-tumor M1-polarization, triple negative breast cancers (TNBCs) promote pro-tumor M2-polarization [24, 25]. Another key player in the microenvironment-promoting breast cancers aggressiveness is endothelial cells (ECs). Vascular endothelial growth factor (VEGF) recruits endothelial progenitors into tumors to promote the transition from micro- to macro-metastases in breast cancers [26]. VEGF/VEGFR signaling has long been the focus of anti-cancer therapies [26]. Tumor microenvironment including the bidirectional interactions with stromal entities, secreted factors, and necrotic, hypoxic and inflammatory conditions within the tumors play a prominent role in enhancing TNBC aggressiveness [27].

BRCA1-IRIS (*aka* IRIS, for **I**n-frame **R**eading of **I**ntron 11 **S**plice variant) is an oncogene produced by the alternative usage of the *BRCA1* locus rather than the alternative splicing of the *BRCA1 mRNA* [28]. While IRIS expression is high in all breast cancer subtypes compared to normal mammary tissue, it is expressed at the highest level in TNBCs [29]. In fact, deliberate IRIS overexpression (IRISOE) in normal mammary epithelial (HME) cells or luminal A/ER^+^ cells converts them into genuine TNBC cells expressing basal-biomarkers, epithelial-to-mesenchymal (EMT)-inducers, and stemness-enforcers, and lacking BRCA1 protein expression *in vitro* and *in vivo* [30, 31]. Moreover, normal HME cells expressing mutant Ras^V12^ or IRISOE develop mammary tumors in SCID mice. However, unlike Ras^V12^-driven tumors, IRISOE-driven tumors contained a large necrotic/hypoxic cores [29], and were more aggressive, implicating the harsh microenvironment within these tumors in their increased aggressiveness. Here, we define the bidirectional interactions with stromal cells that enhance IRISOE TNBC tumor cells aggressiveness. We show an aggressiveness niche, within or near the necrotic/hypoxic/inflamed core of IRISOE tumors, where secreted factors from IRISOE TNBC cells recruit MSCs, TAMs and ECs that cooperate to generate IRISOE TNBC metastatic precursors also through secreted factors.

## RESULTS

### Generation of orthotopic IRISOE mammary tumor cell lines

Generation of TERT-immortalized HME cell lines expressing a doxycycline (Dox)-inducible IRIS allele (IRISOE1-5, Supplementary Figure 1) was described in details earlier [29]. In the absence of Dox these cell lines maintained low-level IRIS, and were referred to as naïve HME. In Dox-containing medium they expressed ∼5 fold higher IRIS [29]. When 5×10^6^ of several of these cell lines were injected into Dox-supplemented (drinking water) SCID mice mammary fat pads, orthotopic mammary tumors developed ∼3 months later (Supplementary Figure 1B). Noteworthy, in the absence of Dox, these naïve HME die, *in vivo*. Detailed analysis of these IRISOE-induced orthotopic mammary tumors was reported recently [32]. These primary (1°) IRISOE mammary tumors were used to generate cell lines now referred to as IRIS291, IRIS292, and IRIS293 (Supplementary Figure 1C). These cell lines maintained Dox-inducible IRIS expression (Supplementary Figure 1D, left) comparable to that observed in several confirmed human TNBC cell lines (Supplementary Figure 1D, right). Additionally, like genuine TNBC cells [29-31], 1° IRISOE mammary tumor cells show high level basal (e.g., CK5, Supplementary Figure 1E-1G), EMT (e.g., vimentin, Supplementary Figure 1H-1J) biomarker expression. Interestingly, as we previously reported IRISOE cells showed high-level expression of CK5 and vimentin (Supplementary Figure 1K-1M), almost completely blocked by IRIS silencing (compare Supplementary Figure 1N to 1K, and 1O to 1L). We refer to these cell lines as orthotopic 1° IRISOE TNBC mammary tumor cell lines.

Orthotopic 1° IRISOE mammary tumors (n>30) contain large necrotic cores (see N in Supplementary Figure 2D, not 2A). Detail analysis of these tumors and necrosis cores was reported recently [29]. Surrounding these necrotic cores within IRISOE (Supplementary Figure 2B, and 2E and inset) tumors are hypoxic cells (see hypoxyprobe staining in Supplementary Figure 2F and inset, not 2C). The uncontrolled release of products from necrotic tumor cells initiates an inflammatory response as well in the surrounding hypoxic cells, which in TNBC tumors is intimately involved in cancer progression [27]. Thus, we wondered whether hypoxic IRISOE TNBC cells produce and secrete inflammatory cytokines [32].

### IL-1β secreted by IRISOE TNBC cells initiates the bi-directional interaction with MSCs

The uncontrolled release of damaged-associated molecular products (DAMPs) from necrotic tumor cells, such as HMGB1 or DNA from the nucleus, uric acid or RNA from the cytoplasm, DNA or ATP from the mitochondria could promote inflammatory responds in the hypoxic tumors cells in the vicinity by binding to several DAMPs receptors, such as RAGE, TLRs and TREM1 [33]. Hypoxia itself can initiate an inflammatory response within tumors, which is intimately involved in cancer progression in TNBC tumors [27].

**Figure 1:**
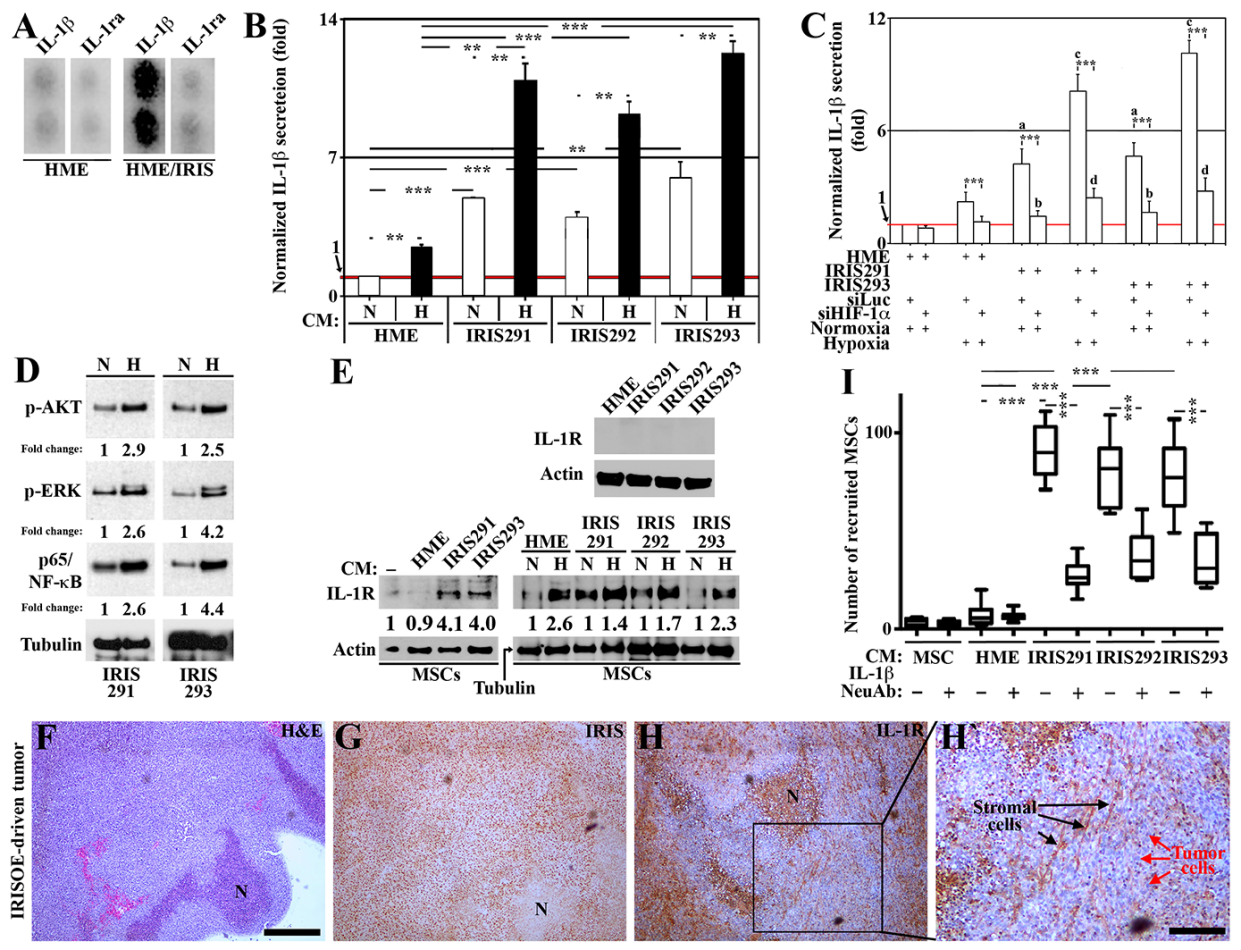
IRISOE TNBC cells secrete IL-1 β to recruit and activate MSCs **(A)** IL-1β and IL-1ra levels in HME cells transfected with doxycycline-inducible IRIS allele in the absence (HME) or presence of 2μg/ml of Dox (72h, HME/IRIS). **(B)** Normalized IL-1β level detected using ELISA in the conditioned medium (CM) of HME, IRIS291, IRIS292, or IRIS293 cells grown under normoxic (N) or hypoxic (H) conditions for 24h. **(C)** Normalized IL-1β level detected using ELISA in the CM of HME, IRIS291, or IRIS293 cells transfected with siLuc or siHIF-1α for 48h, followed by growth in N or H conditions for an additional 24h. **(D)** Western blot analysis for the level of activated AKT, ERK, or NF-κB/p65 in IRIS291, or IRIS293 cells grown under N or H conditions for 24h. **(E)** Western blot analysis of the surface expression of IL-R in naïve HME, IRIS291-IRIS293 (upper), naïve MSCs grown for 24h in the absence [-] or presence of CM from naïve HME, IRIS291, IRIS292, or IRIS293 pre-exposed or not to hypoxic conditions (lower). **(F-H)** IHC analysis of the expression of IRIS, and IL-1R in a 1° orthotopic IRISOE mammary tumor. (H) Higher magnification image of the area squared in I. Scale bar: 500μm in F-H, and 100μm in H`. **(I)** Recruitment of MSC towards IRISOE cells CM in the absence or presence of an IL-1β NeuAb analyzed using Boyden chamber.

Whether hypoxic IRISOE TNBC cells produce and secrete inflammatory cytokines was sought next [32]. A recent antibody array showed that compared to naïve HME cells conditioned medium (CM), IRISOE cells CM contained high-levels of several cytokines, chemokines, and growth factors (not shown). Among these cytokines was the most prominent inflammatory cytokine; IL-1β (Figure 1A). Interestingly, both naïve and IRISOE cells CM contained very low-levels of the endogenous IL-1β inhibitor; IL-1ra (Figure 1A). Moreover, the level of IL-1β secreted from the TNBC cell lines; MDA-MB-231 (MDA231) and MDA-MB-468 (MDA468) is 3-4 fold higher than that secreted from the non-TNBC/luminal A cell lines; MCF7 and T47D (not shown). IL-1β secretion decreased >50% upon IRIS silencing in MDA231 and MDA468 (red bars, Supplementary Figure 3, left) [34], and increased ∼2 fold when IRIS was overexpressed in MCF7 and T47D (red bars, Supplementary Figure 3, right) [35].

Next, we assessed whether hypoxia plays a role in IL-1β secretion from IRISOE cells. Using a co-culture protocol (see details, Supplementary Figure 4) followed by ELISA analysis that measured low-level of IL-1β in normoxic naïve HME cells CM, taken as 1 (N and red line, Figure 1B), increased slightly but significantly upon hypoxia in these cells (Figure 1B). Between 4-6 fold higher levels of IL-1β were measured in IRIS291, IRIS292, and IRIS293 cells CM (N, Figure 1B), exacerbated upon hypoxia to 11-13 fold higher (H, Figure 1B). To test the role of HIF-1α in this hypoxia-induced IL-1β secretion from IRISOE cells. Naïve HME, IRIS291, and IRIS293 were transfected with luciferase (siLuc) or HIF-1α siRNA (siHIF-1α) for 48h before they were exposed to normoxic or hypoxic conditions for an additional 24h. According to ELISA, HIF-1α-silencing significantly blocked hypoxia-induced IL-1β secretion from all cell lines (Figure 1C). However, HIF-1α-silencing also blocked IL-1β secretion from normoxic IRISOE cells (Figure 1C), suggesting that IRISOE stabilizes HIF-1α (or increase its expression) under normoxic condition. To verify that experimentally, we used Western blot, which showed that compared to hypoxic naïve HME cells, the level of HIF-1α is 2-3 fold higher in normoxic IRIS291, or IRIS293 cells (compare lane 1 and 3, respectively to 2, Supplementary Figure 5). Moreover, IRIS silencing in normoxic MDA-MB-231 (compare lane 5 to 6 in Supplementary Figure 5) or in normoxic MDA-MB-468 (compared lane 9 to 8, Supplementary Figure 5) significantly decreased HIF-1α levels. Together confirm that IRISOE stabilizes (although we cannot rule out an effect on expression) HIF1α under normal/normoxic condition leading to enhance in IL-1β expression/secretion. Hypoxic condition further exacerbates this production/secretion in all cells (even naïve HME).

Previously it was shown that HIF-1α activates downstream signaling involved in IL-1β expression/secretion [34]. Thus, using Western blot, we measured the levels of activated AKT, ERK and most importantly NF-κB in normoxic vs. hypoxic IRIS291 and IRIS293 cells. Hypoxic (24h) IRIS291 cells total proteins (isolated by sonication of whole cells) contained 2.9, 2.6, and 2.6 fold higher p-AKT and p-ERK1/2 (i.e. activated), and p65/NF-κB accumulation (i.e. activation), respectively, compared to normoxic IRIS291 cells total proteins (compare H to N, Figure 1D, left). Similarly, hypoxic IRIS293 cells total proteins contained 2.5, 4.2, and 4.4 fold higher p-AKT, p-ERK1/2, and p65/NF-κB, respectively, compared to normoxic IRIS293 cells total proteins (compare H to N, Figure 1D, right). Noteworthy, activated AKT, ERK, or NF-κB activate HIF-1α signaling in TNBC cells, leading to IL-1β expression/secretion [34]. Together suggest IRISOE enhances production/secretion of IL-1β in TNBC tumor cells under normal/normoxic, as well as hypoxic (e.g., within the aggressiveness niche in IRISOE TNBC tumors [35]) conditions through stabilization of HIF-1α and/or activation of AKT, ERK and/or NF-κB signaling.

TNBC cells establish contact with MSCs within tumors using IL-1β [35]. Thus, whether IRISOE TNBC cells interact with MSCs through IL-1β was investigated. First, we found using Western blot that neither naïve HME, nor IRIS291, IRIS292, IRIS293 cells express the receptor for IL-1β; IL-1R on their surface (isolated cell membranes, Figure 1E, upper). Following the co-culture protocol outlined in Supplementary Figure 4, we then measured also using Western blot the level of IL-1R on the surface of naïve MSCs or those exposed to naïve HME or IRISOE cells CM (see Supplementary Figure 4). Naïve MSCs do not express IL-1R on their surface ([-] Figure 1F, lower left,). Naïve MSCs exposed to naïve HME CM (24h) also did not show IL-1R on their surface (Figure 1E, lower left). In contrast, naïve MSCs exposed to IRIS291 or IRIS293 CM (24h) showed ∼4 fold increase in IL-1R level on their surface (Figure 1E, lower left). Interestingly, the level of IL-1R on naïve MSCs surface increased even further if they were exposed (24h) to IRISOE cells CM (even naïve HME cells CM) that were prior exposed (24h) to hypoxic conditions (Figure 1E, lower right). Moreover, IHC staining of adjacent sections from 1° IRISOE orthotopic mammary tumors showed that although necrotic areas (see N, Figure 1F) were devoid of IRIS (see N, Figure 1G) they expressed high level of IL-1R (see N, Figure 1H), supporting enhanced expression of IL-1R on the surface of stromal and not necrotic mammary cells. Additionally, according to high-magnification image of the IL-1R staining only elongated stromal cells (e.g., MSCs) stained for IL-1R (black arrows Figure 1H), whereas epithelial cells were IL-1R negative (red arrows, Figure 1H). Finally, to establish this even further, the same tumor was fluorescently IHC stained with IL-1R and the mouse MSCs specific cell surface marker, CD90 (although it is also expressed by other cells of hematopoietic origin) [36, 37]. Only CD90^+^ cells (Supplementary Figure 6C and 6D) co-stained with IL-1R (Supplementary Figure 6B and 6D). Together suggest that IL-1β secreted by IRISOE TNBC tumor cells induces expression of its own receptor IL-1R on the surface of the negative naïve MSCs.

Next, green fluorescent protein (GFP)-expressing MSCs were layered in inserts of Boyden chambers (8μm pore size) and naïve MSCs, naïve HME, IRIS291, IRIS292, and IRIS293 cells CM were placed in the lower chambers in the presence or absence of IL-1β neutralizing antibody (IL-1β NeuAb). Naïve MSC and naïve HME cells CM attracted insignificant numbers of GFP-MSC to their vicinity and that was not affected by the IL-1β NeuAb addition (Figure 1I). In contrast, IRIS291, IRIS292 and IRIS293 cells CM attracted massive numbers of MSCs to their vicinities, an effect that was significantly blocked by the IL-1β NeuAb (Figure 1I). Together suggest that at least in culture, IL-1β secreted by IRISOE TNBC tumor cells recruits MSCs to the vicinity of tumor cells, most likely through inducing expression of IL-1R on naïve MSCs surface.

**Figure 2:**
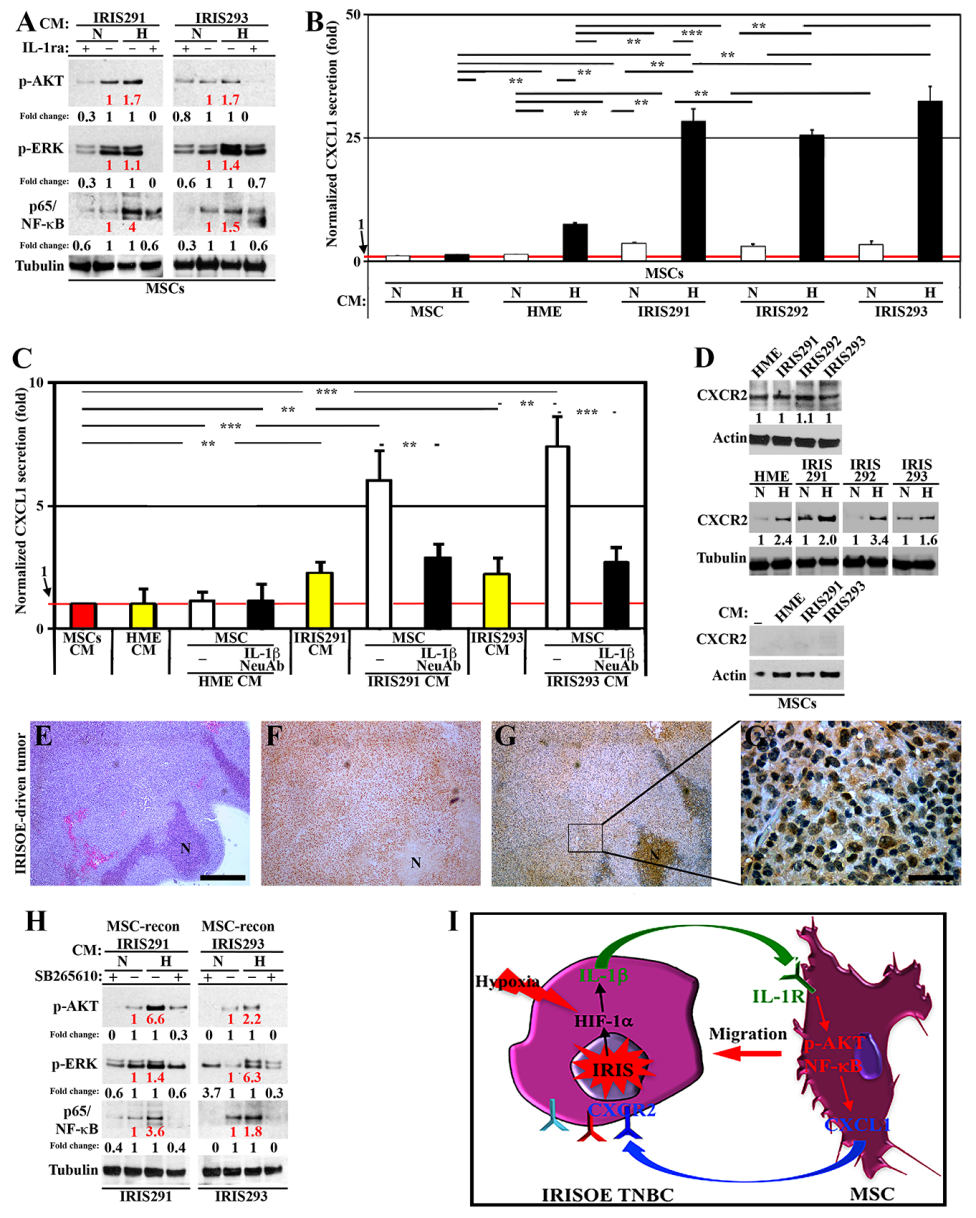
MSCs respond by secreting CXCL1 to re-activate IRISOE TNBC cells **(A)** Expression of activated AKT, ERK and NF-κB/p65 in MSCs exposed 24h to CM from normoxic (N) or hypoxic (H, 24h) IRIS291 or IRIS293 cells in the presence or absence of IL-1ra. **(B)** Normalized CXCL1 level detected using ELISA in the CM of naïve MSCs exposed (24h) to CM from naïve MSCs, HME, IRIS291, IRIS292, or IRIS293 cells pre-exposed to N or H (24h). **(C)** Normalized level of CXCL1 level detected using ELISA in CM of naïve MSCs (red bar), HME, IRIS291, IRIS293 alone (yellow bars), or MSCs incubated with HME, IRIS291, or IRIS293 CM supplemented with vehicle (white bars) or IL-1β NeuAb (black bars) for 24h. **(D)** The level of CXCR2 expressed on the surface of HME, IRIS291, IRIS292, or IRIS293 grown in N or H (24h, upper), or MSCs in the absence [-], or exposed (24h) to HME, IRIS291, or IRIS293 CM (lower). Note, actin blots are the same as in Figure 1E, and that this experiment and the one in Figure 1E represent positive controls to one another. **(E-G)** IHC analysis for CXCR2 expression in 1° IRISOE orthotopic tumor. (G) Higher magnification image of the area squared in G. Scale bar: 500μm in E-G, and 50μm in (G`). **(H)** Western blot analysis of the expression of activated AKT, ERK, NF-κB/p65 in IRIS291, IRIS293 cells following exposure to corresponding normoxic or hypoxic (24h) mammary cells CM reconditioned by MSCs contact (24h) in the absence or presence (24h) of SB265610. **(I)** Proposed model for the reciprocal interaction between IRISOE cells and MSCs.

Next, to evaluate the functional significant of IL-1β secretion, we again focused on the interaction with MSCs. Normoxic or hypoxic (24h) naïve HME, IRIS291 or IRIS293 cells CM was added (24h) to naïve MSC in the presence or absence of IL-1ra. Western blot was then used on protein isolated by sonication of whole cells (see experimental details in Supplementary Figure 4). Compared to naïve MSCs exposed to normoxic IRIS291 cells CM, those exposed to hypoxic IRIS291 cells CM contained 1.7, 1.1, and 4 fold higher activated AKT, ERK, and p65/NF-κB (red numbers, Figure 2A, left). Similarly, compared to naïve MSCs exposed to normoxic IRIS293 cells CM, those exposed to hypoxic IRIS293 cells CM contained 1.7, 1.4, and 1.5 fold higher activated AKT, ERK, and p65/NF-κB (red numbers, Figure 2A, right). IL-1ra inhibited activation of these factors between 40-100% whether MSCs were incubated with normoxic (compare lanes 1 to 2 in left and right, Figure 2A) or hypoxic (compare lanes 4 to 3 in left and right, Figure 2A) tumor cells CM. Together suggest that at least in culture, after recruiting MSCs to the vicinity of tumor cells, IL-1β secreted by IRISOE TNBC tumor cells through IL-1R activation, it activates AKT, ERK, and NF-κB signaling within these naïve MSCs.

Finally, aggressive breast cancer cells stimulate secretion of CXCL1, 6 and 8 from MSCs [38]. Our preliminary experiments revealed that when exposed to IRISOE CM, MSCs secrete elevated level of CXCL1 (not 6 or 8). Focusing on CXCL1, CM from MSCs exposed to normoxic or hypoxic (24h) naïve MSCs, naïve HME, IRIS291, IRIS292, or IRIS293 cells CM (see experimental details in Supplementary Figure 4), were examined by ELISA. MSCs, whether exposed to normoxic or hypoxic naïve MSCs CM, secreted very low-level CXCL1 (taken as 1, red line Figure 2B). Hypoxic not normoxic naïve HME cells CM induced CXCL1 secretion from MSC (compare black to white bar, Figure 2B), whereas normoxic (white bars, Figure 2B) as well as hypoxic (black bars, Figure 2C) IRIS291, IRIS292, or IRIS293 cells CM induce CXCL1 secretion from MSCs. Moreover, according to ELISA, CM from naïve MSCs exposed (24h) to naïve MSCs CM showed very low-level CXCL1 (taken as 1, red bar and line, Figure 2C). Compared to naïve HME cells, IRIS291 or IRIS293 cells themselves secrete low-level CXCL1 (yellow bars, Figure 2C). Compared to CM from naïve MSCs exposed (24h) to naïve HME cells CM, CM from those exposed (24h) to IRIS291 or IRIS293 cells CM showed high-level CXCL1 (white bars, Figure 2C) that was significantly blocked when IL-1β NeuAb was included (black bars, Figure 2C). Together suggest that IL-1β secreted from IRISOE tumor cells upregulates expression of its own receptor; IL-1R on the surface of naïve MSCs only, leading to activation of AKT, ERK, and p65/NF-κB signaling within MSCs, which leads to secretion of CXCL1 from MSCs. All these effects are exacerbated by hypoxia, most likely within the aggressiveness niche, *in vivo*. Thus, IL-1β effect seems to be unidirectional from IRISOE tumor cells to MSCs.

Western blot analysis showed that the receptor for CXCL1; CXCR2 is expressed at high levels on naïve HME, as well as IRIS291, IRIS292, and IRIS293 cells surface (Figure 2D, upper left). Interestingly, expression of CXCR2 on IRISOE (even naïve HME) cells was exacerbated by hypoxia (Figure 2D, middle). In contrast, naïve MSC, or naïve MSC exposed to naïve HME, IRIS291, or IRIS293 cells CM did not show any expression of CXCR2 (Figure 2D, lower). Furthermore, IHC analysis of 1° IRISOE orthotopic mammary tumor confirmed that high-level CXCR2 could be observed on the surface of tumor cells only (Figure 2E-2G). Taken together suggest that whether secreted by IRISOE cells or MSCs exposed to IRISOE CM, CXCL1 signaling is unidirectional from MSCs to IRISOE tumor cells.

Finally, normoxic or hypoxic (24h) IRIS291 or IRIS293 cells CM was added to naïve MSC (24h), before it was re-added to the same IRISOE cell line (24h) in the absence or presence of the CXCR2 specific inhibitor “SB265610” (see experiment details, Supplementary Figure 4). Western blot on sonicated cell extracts showed that IRIS291 cells exposed to their own hypoxic CM reconditioned by MSCs contact expressed 6.6, 1.4, 3.6 higher activated AKT, ERK1/2, and p65/NF-κB compared to normoxic CM reconditioned by MSCs contact (red numbers, Figure 2H, left). IRIS293 cells exposed to their own hypoxic CM reconditioned by MSCs contact expressed 2.2, 6.3, 1.8 higher activated AKT, ERK1/2, and p65/NF-κB compared to normoxic CM reconditioned by MSCs contact (red numbers, Figure 2H, right). Including the CXCR2 inhibitor “SB265610” before re-addition to either IRISOE cells significantly decreased the levels of these activated proteins within the tumor cells whether the original CM was from normoxic (compare lanes 1 to 2 in left and right, Figure 2H) or hypoxic (compare lanes 4 to 3 in left and right, Figure 2H) IRISOE cells. The data so far suggest that *in vivo* within the aggressiveness niche, secretion of IL-1β by IRISOE cells is exacerbated by hypoxia, and acts in paracrine fashion to elevate expression of IL-1R on the surface of naïve MSCs, recruits them to the vicinity of tumor cells in the niche, activates AKT, ERK, and NF-κB signaling in them, leading to production/secretion of CXCL1 from MSCs, which also in paracrine fashion activates IRISOE tumor cells (see model in Figure 2I).

### CCL2 secreted by MSCs-entrained IRISOE TNBC cells initiates the bi-directional interaction with TAMs

**Figure 3:**
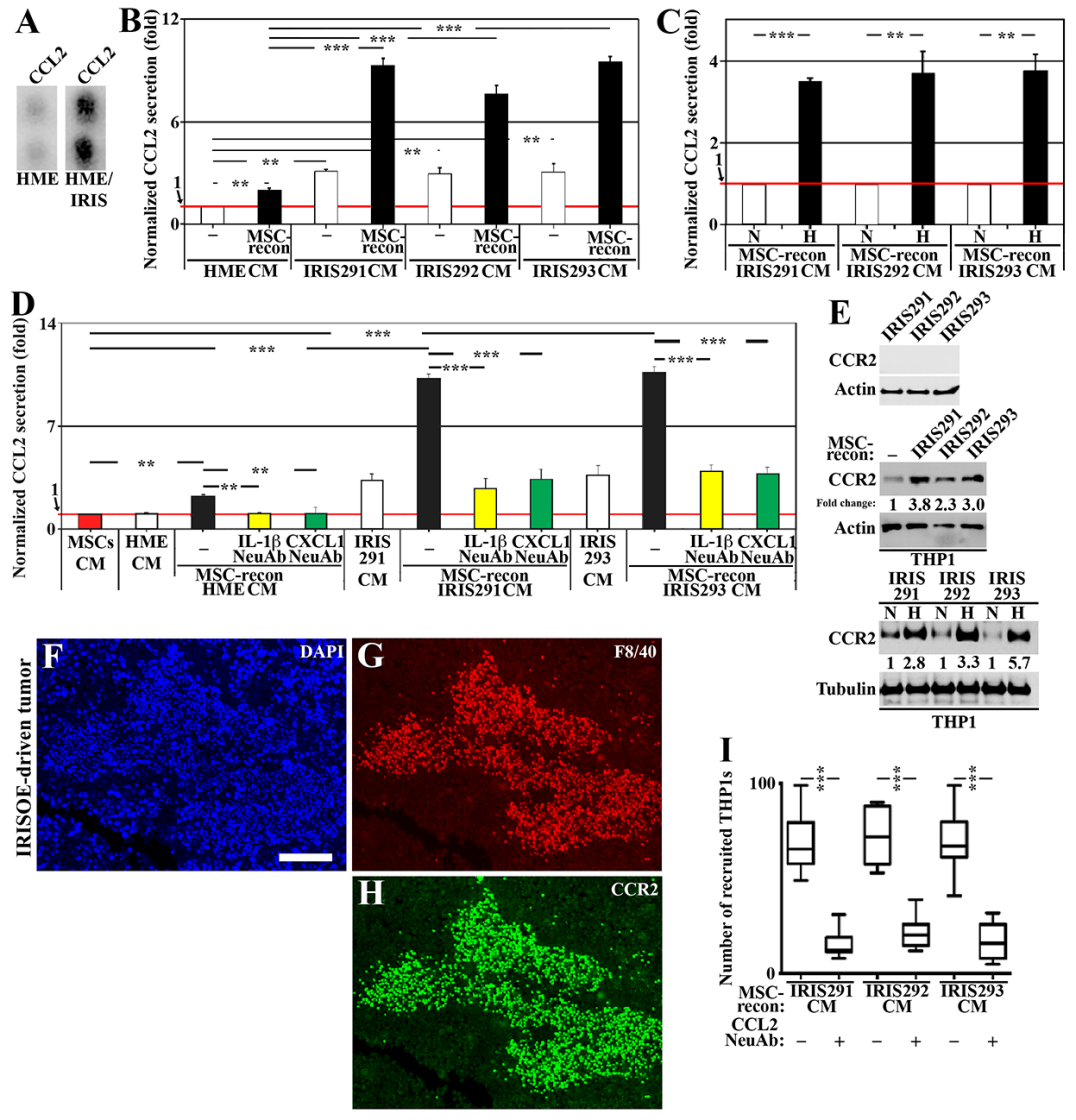
IRISOE cells entrained by MSCs recruit and activate TAMs **(A)** CCL2 level in HME cells transfected with doxycycline-inducible IRIS allele in the absence (HME) or presence of 2μg/ml of Dox (72h, HME/IRIS). **(B)** Normalized level of CCL2 level detected by ELISA in CM of HME, IRIS291, IRIS292, or IRIS293 cells or CM from these cells reconditioned (24h) by MSCs contact. **(C)** Normalized CCL2 level detected by ELISA in CM of normoxic or hypoxic IRIS291, IRIS292 or IRIS293 cells reconditioned (24h) by MSCs contact. **(D)** Normalized level detected by ELISA of CCL2 secreted from naïve MSCs (red bar), HME, IRIS291, or IRIS293 alone (white bars), or in CM from HME, IRIS291, or IRIS293 reconditioned by MSCs contact in the absence (black bars) or presence of IL-1β NeuAb (yellow bars) added before MSCs contact, or CXCL1 NeuAb (green bars) added after MSCs contact. **(E)** Western blot analysis of CCR2 level on the surface of IRIS291, IRIS292, and IRIS293 cells (upper), or the surface of THP1-macrophages unexposed [-] or exposed to IRIS291, IRIS292, or IRIS293 CM reconditioned by MSCs contact (24h, middle), and same assay performed with normoxic or hypoxic original mammary cells CM (lower). **(F-H)** Fluorescent IHC staining for CCR2 and the mouse macrophage specific marker F4/80 in 1º IRISOE orthotopic tumor. Scale bars: 200μm in F-H. **(I)** Recruitment of THP1-macrophages towards CM from IRIS291, IRIS292 or IRIS293 cells reconditioned by MSC contact (24h) in the absence or presence of CCL2 NeuAb detected using Boyden chambers.

In the same antibody array described above, we found that IRISOE cells CM contained higher level of CCL2 than naïve HME cells CM (Figure 3A). Moreover, the level of CCL2 secreted from MDA231 and MDA468 is 2-3 fold higher than that secreted from MCF7 and T47D (not shown). CCL2 secretion decreased by 40-50% upon IRIS silencing in MDA231 and MDA468 (white bars, Supplementary Figure 3, left), and increased by 50-60% when IRIS was overexpressed in MCF7 and T47D (white bars, Supplementary Figure 3, right).

Whether hypoxia and/or MSCs interaction play a role in the enhanced secretion of CCL2 from IRISOE TNBC cells was sought next. CM from normoxic or hypoxic (24h) naïve HME, IRIS291, IRIS292, or IRIS293 cells was reconditioned by MSCs contact (24h) before it was re-added to the same cell line (24h, see experimental details, Supplementary Figure 4) followed by ELISA. Without MSC contact, IRIS291, IRIS292, and IRIS293 secrete ∼2 fold higher of CCL2 compared to naïve HME cells (white bars, Figure 3B). After MSC contact, naïve HME cells began to secrete CCL2, and IRIS291, IRIS292, and IRIS293 began to secrete 6-8 fold CCL2 (black bars, Figure 3B). If the original CM was from hypoxic IRIS291, IRIS292, or IRIS293 cells before it was reconditioned by MSC contact, it induced even higher CCL2 secretion when re-added to IRISOE TNBC cells (compare black to white bars, Figure 3C). Finally, naïve MSCs secrete low-level CCL2 (taken as 1, red bar and line, Figure 3D), whereas IRIS291 and IRIS293 cells (not naïve HME cells) secrete high-level CCL2 (white bars, Figure 3D). Naïve HME cells exposed to naïve HME cells CM reconditioned by MSC contact secreted slightly higher level of CCL2 (compare black to white bar, Figure 3D). Additionally, IRIS291 and IRIS293 cells exposed to their CM reconditioned by MSC contact secreted much higher levels of CCL2 (compare white to black bars, Figure 3D). However, including IL-1β NeuAb before MSCs contact (yellow bars, Figure 3D) or CXCL1 NeuAb after MSCs contact (green bars, Figure 3E) significantly blocked CCL2 secretion from all cell lines. Together suggest that while IRISOE tumor cells secrete low-level CCL2 under normal condition, hypoxia and/or MSCs contact through the IL-1β/CXCL1 circuit exacerbates CCL2 secretion from these cells.

Next, we investigated the biological effect of CCL2 secreted from IRISOE tumor cells on macrophages. For this task, we used THP1 cell line, which is a good model to study macrophage biology *in vitro* [39]. THP1 is a primary monocyte cell line that differentiates into non-polarized macrophages when incubated with phorbol myristate acetate (PMA) for 4 days (hereafter THP1-macrophages).

Western blot revealed that IRIS291, IRIS292, and IRIS293 cells do not express the CCL2 receptor; CCR2 on their surface (Figure 3E, upper). On the other hand, naïve THP1-macrophages express low-level CCR2 on their surface ([-], Figure 3E, middle). CCR2 expression on THP1-macrophages surface significantly increased following exposure to IRIS291, IRIS292, or IRIS293 cells CM, reconditioned by MSCs contact (24h) then by IRISOE cells contact (24h, Figure 3E, middle). Interestingly, if the original IRISOE tumor cells CM was from hypoxic cells even further increase in CCR2 expression on the surface of THP1-macrophages was observed (Figure 3E, lower). Additionally, according to fluorescent IHC staining of the 1° IRISOE mammary tumors only F4/80^+^ (mouse macrophage-specific biomarker) cells are CCR2^+^ (Figure 3F-3H). Together suggest that CCL2 secreted by MSCs-entrained IRISOE TNBC tumor cells induces expression of its own receptor CCR2 on the surface of the low expressing THP1-macrophages.

Next, we layered equal number of THP1-macrophages on inserts of 8μm pore size Boyden chambers. Inserts were then exposed (24h) to IRIS291, IRIS292, or IRIS293 cells CM re-conditioned by MSCs contact (24h) then by IRISOE cells contact (24h) in the absence or presence of CCL2 NeuAb (see experimental details, Supplementary Figure 4). While large number of THP1-macrophages migrated towards all CM in the absence of the CCL2 NeuAb, the numbers significantly decreased in the presence of the CCL2 NeuAb (Figure 3I). Together suggest that at least in culture, CCL2 secreted by MSCs-entrained IRISOE TNBC tumor cells recruits THP1-macrophages to the vicinity of tumor cells, most likely through inducing expression of CCR2 on naïve THP1-macrophages surface.

**Figure 4:**
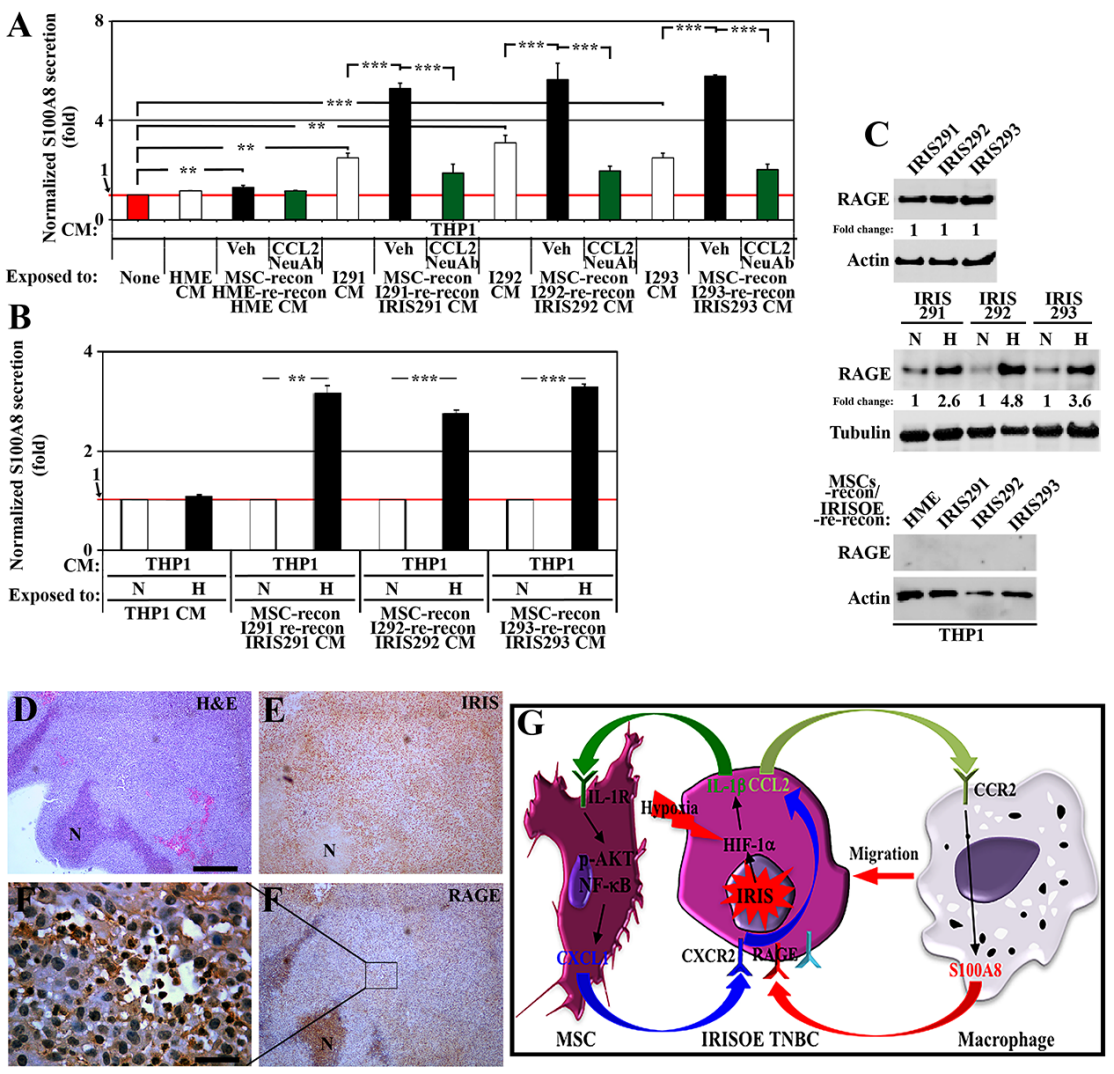
TAMs respond by secreting S100A8/9 to re-activate IRISOE TNBC cells **(A)** Normalized S100A8 level detected by ELISA in the CM of THP1-macrophages unexposed (red bar) or exposed to HME, IRIS291, IRIS292 or IRIS293 CM (white bars), or same cells CM reconditioned by MSCs contact (24h) then reconditioned by the same mammary cell line contact (24h), in the absence (black bars) or presence of CCL2 NeuAb (green bars). **(B)** Normalized S100A8 level detected by ELISA in the CM of THP1-macrophages exposed to naïve THP1 CM, or CM from normoxic (white bars), or hypoxic (24h, black bars) IRIS291, IRIS292, or IRIS293 cells reconditioned by MSCs contact (24h). **(C)** The expression of RAGE on the surface of normoxic, or hypoxic (24h) IRIS291, IRIS292, or IRIS293 cells (upper), or THP1 exposed to CM from naïve HME, IRIS291-IRIS291 cells reconditioned by MSC contact (24h), then by the same mammary cell line contact (24h, lower). **(D-F)** IHC analysis of RAGE expression in 1° IRISOE orthotopic tumor. (F`) Higher magnification image of the area squared in F. Scale bars: 500μm in (D-F), and 50μm in F`. **(G)** Proposed model for the signaling axis between MSCs-entrained IRISOE TNBC cells and TAMs. Note, actin blots are the same as in Figure 3E, and that this experiment and the one in Figure 3E represent positive controls for one another.

TAMs promote immunosuppression by secreting pro-inflammatory proteins; such as the calcium- and zinc-binding protein, S100A8/9, which plays a prominent role in the regulation of inflammatory processes and immune response [40]. To investigate the role TAMs, play in enhancing IRISOE TNBC cells aggressiveness, normoxic or hypoxic (24) naïve HME or IRISOE tumor cells CM was re-conditioned by MSC (24h) then by the same cell line contact (24h) before it was added onto THP1-macrophages (24h, for details, see Supplementary Figure 4). ELISA analysis of these CM revealed low-level S100A8 is secreted by THP1-macrophages (taken as 1, red bar and line, Figure 4A). Unlike naïve HME cells, IRIS291, IRIS292, and IRIS293 induced secretion of S100A8 from THP1-macrophages (white bars, Figure 4A). MSCs contact enhanced naïve HME cells CM and exacerbated IRISOE tumor cells CM ability to induce THP1-macrophages to secrete S100A8 (compare black to white bars, Figure 4A). Finally, including CCL2 NeuAb blocked the ability to these CM to induce THP1-macrophages to secrete S100A8 (compare green to black bars, Figure 4A). Importantly, if the original IRISOE TNBC tumor cells CM was from hypoxic cells it further induced S100A8 secretion from THP1-macrophages (Figure 4B). Together suggest that although IRISOE tumor cells normally secrete CCL2, entrainment by hypoxia and/or MSC contact exacerbates the secretion leading to recruitment of macrophages to the vicinity of IRISOE tumor cells, most likely within the aggressiveness niche, *in vivo*, through upregulating the expression of CCR2 on macrophages, and activating them to secrete S100A8. Thus, CCL2 effect seems to be unidirectional from IRISOE tumor cells to macrophages.

RAGE is the major S100A8 receptor [41]. Normoxic or hypoxic (24h) naïve HME or IRISOE cells CM was re-conditioned by MSC contact (24h) then by same cell line contact (24h) before it was added on THP1-macrophages (24h) and Western blot analysis was performed on membrane fraction (see experimental details, Supplementary Figure 4). IRIS291, IRIS292, and IRIS293 cells express high levels of RAGE on their surface (Figure 4C, upper) that increased even further under hypoxic conditions (Figure 4C, middle). In contrast, THP1-macrophages, whether exposed to naïve HME or IRISOE tumor cells CM re-reconditioned by MSC then IRISOE tumor cells show no RAGE expression on their surface (Figure 4C, lower). Indeed, according to IHC staining of orthotopic 1° IRISOE-driven tumors, only tumor cells express RAGE (Figure 4D-4F). Together suggest that IL-1β secreted by IRISOE tumor cells activates MSCs in a paracrine fashion to secrete CXCL1, which also in a paracrine fashion activates IRISOE tumor cells to secrete CCL2. CCL2, in a paracrine fashion recruits macrophages, most likely to the aggressiveness niche, *in vivo*, and activates them to secrete S100A8/9. Hypoxia exacerbates all the steps even CCR2 expression on macrophages and RAGE on IRISOE tumor cells (see Figure 4G).

### VEGF secreted by MSCs-entrained IRISOE TNBC cells initiates the bi-directional interaction with ECs

**Figure 5:**
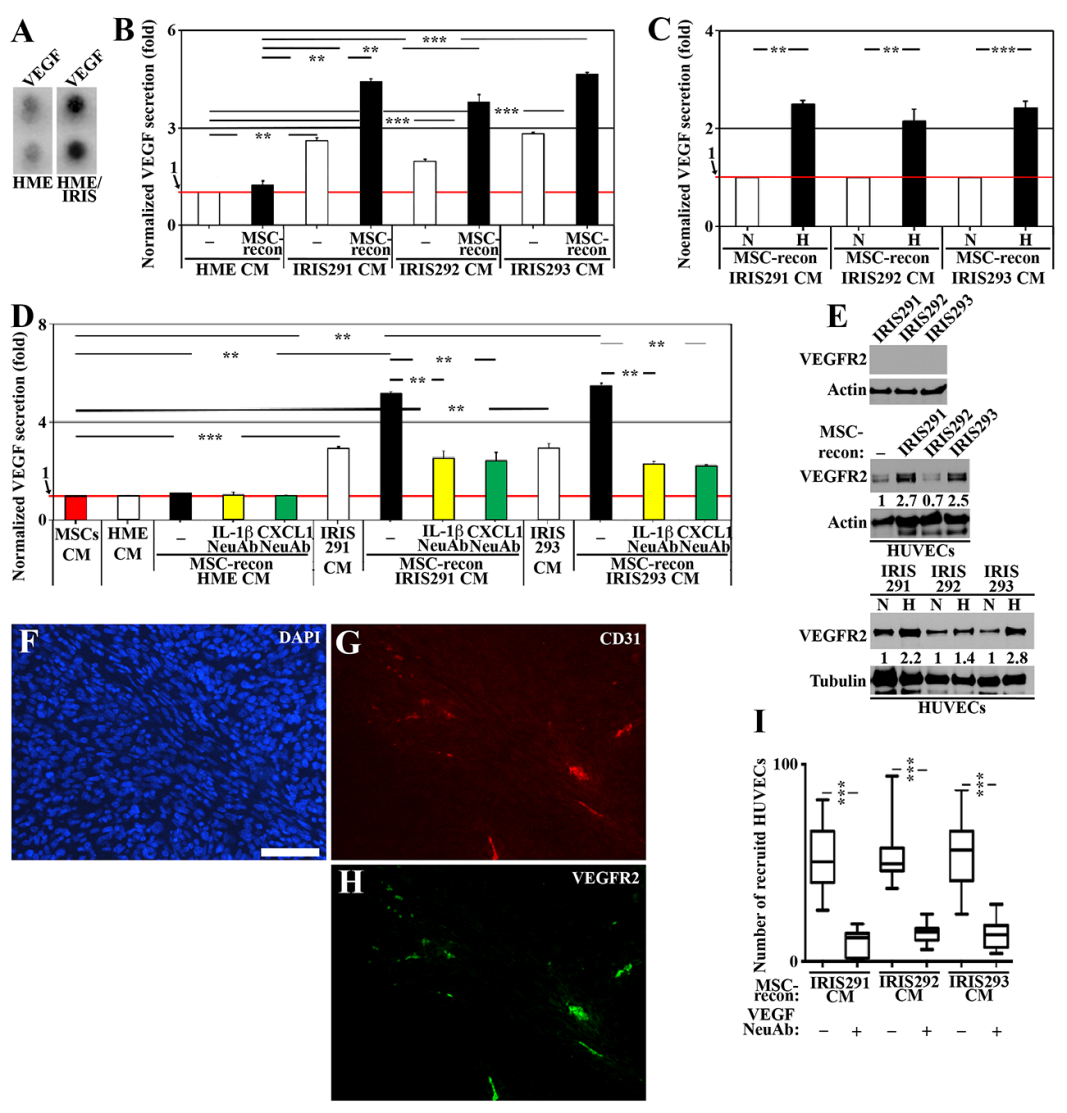
IRISOE cells entrained by MSCs recruit and activate ECs **(A)** VEGF level in HME cells transfected with doxycycline-inducible IRIS allele in the absence (HME) or presence of 2μg/ml of Dox (72h, HME/IRIS). **(B)** Normalized VEGF level detected by ELISA in CM from HME, IRIS291, IRIS292 or IRIS293 cells, or CM from these cells reconditioned (24h) by MSCs contact. **(C)** Normalized VEGF level detected by ELISA in CM from normoxic or hypoxic IRIS291, IRIS292 or IRIS293 cells reconditioned (24h) by MSCs contact. **(D)** The level of VEGF detected by ELISA secreted from naïve MSCs (red bar), HME, IRIS291 or IRIS293 alone (white bars), or in CM from HME, IRIS291 or IRIS293 reconditioned by MSCs contact in the absence (black bars) or presence of IL-1β NeuAb (yellow bars) added before MSCs contact, or CXCL1 NeuAb (green bars) added after MSCs contact. **(E)** Western blot analysis of VEGFR2 level on the surface of IRIS291, IRIS292, and IRIS293 cells (upper), or the surface of HUVEC unexposed [-] or exposed to IRIS291, IRIS292 or IRIS293 CM reconditioned by MSCs contact (24h, middle), and the same assay performed with normoxic or hypoxic original mammary cells (lower). **(F-H)** Fluorescent IHC staining for VEGFR2, and the mouse endothelial cells specific marker CD31 in 1° IRISOE orthotopic tumor. Scale bars: 100μm in F-H. **(I)** Recruitment of HUVEC towards CM from IRIS291, IRIS292, or IRIS293 cells reconditioned by MSC contact (24h) in the absence or presence of VEGF NeuAb detected using Boyden chambers.

VEGF role in tumor neo-angiogenesis is well documented [42]. In the antibody array described above, we observed also that IRISOE cells CM contained higher level of VEGF compared to naïve HME cells CM (Figure 5A). Moreover, the level of VEGF secreted from MDA231 and MDA468 is >2 fold higher than that secreted from MCF7 and T47D (not shown). VEGF secretion decreased by >50% upon IRIS silencing in MDA231 and MDA468 (black bars, Supplementary Figure 3, left), and increased by >50% when IRIS was overexpressed in MCF7 and T47D (black bars, Supplementary Figure 3, right).

Whether hypoxia and/or MSCs interaction play a role in the enhanced secretion of VEGF from IRISOE TNBC cells was sought next. Normoxic or hypoxic (24h) naïve HME or IRISOE cells CM was reconditioned by MSCs contact (24h) in the absence or presence of IL-1β NeuAb before it was re-added to the same cell line (24h) in the absence or presence of CXCL1 NeuAb followed by ELISA analysis (for experimental details, see Supplementary Figure 4). Compared to naïve HME cells CM, IRIS291, IRIS292, and IRIS293 CM contained higher levels of VEGF (white bars, Figure 5B, C). MSCs contact did not significantly affect VEGF secretion from naïve HME cells, while significantly enhanced VEGF secretion from all IRISOE tumor cells (compare black to white bars, Figure 5B, 5C). Moreover, if the original IRISOE CM was from hypoxic cells, VEGF secretion increased even higher from these cells (Figure 5C). Finally, naïve MSCs secrete very low-level VEGF (taken as 1, red bar and line, Figure 5D). While naïve HME cells also secrete very low-level VEGF, IRISOE cells secrete relatively high-level VEGF (white bars, Figure 5D). Importantly, MSCs reconditioning, while did not affect secretion from naïve HME cells, significantly enhanced secretion from IRISOE cells (black bars, Figure 5D). Addition of IL-1β NeuAb before MSCs contact (yellow bars, Figure 5D), or CXCL1 NeuAb after MSCs (green bars, Figure 5D) abrogated that increase in VEGF secretion from IRISOE tumor cells. Together suggest that while IRISOE tumor cells secrete low-level VEGF under normal condition, hypoxia and/or MSCs contact through the IL-1β/CXCL1 circuit exacerbates VEGF secretion from these cells.

Next, we investigated the biological effect of VEGF secreted from IRISOE tumor cells on ECs. The human umbilical vein endothelial cell line (HUVEC) is a good model to study angiogenesis, *in vitro* [43]. IRIS291, IRIS292, and IRIS293 express no VEGFR2 (the predominant form in VEGFR expressed on TNBC cells [44]) on their surface (Figure 5E, upper). Naïve HUVECs express low-level VEGFR2 on their surface ([-], Figure 5E, middle), increased even further when exposed (24h) to IRISOE cells CM reconditioned by MSC contact (24h), then same cell line contact (24, Figure 5E, middle). This induction was increased even further if the original IRISOE CM was from hypoxic cells (Figure 5E, lower). Accordingly, fluorescent IHC staining of orthotopic 1° IRISOE-driven mammary tumor sections confirmed that only CD31^+^ (specific biomarker of mouse endothelial cells “ECs”) cells are VEGFR2^+^ (Figure 5F-5H). Together suggest that VEGF secreted by MSCs-entrained IRISOE TNBC tumor cells induces expression of its own receptor VEGFR2 on the surface of the low expressing HUVECs.

We layered equal numbers of naïve HUVECs on Boyden chambers inserts (8μm pore size). Inserts were incubated with IRIS291, IRIS292 or IRIS293 cells CM (24h) re-conditioned by MSCs contact (24h), then by same IRISOE cell line contact (24) added in the lower chambers in the presence or absence of VEGF NeuAb (24h, for experimental details see Supplementary Figure 4). Large numbers of HUVECs were recruited to each CM in the absence but significantly dropped in the presence of VEGF NeuAb (Figure 5I). Together suggest that at least in culture, VEGF secreted by MSCs-entrained IRISOE TNBC tumor cells recruits HUVECs to the vicinity of tumor cells, most likely through inducing expression of VEGFR on naïve HUVECs surface.

**Figure 6:**
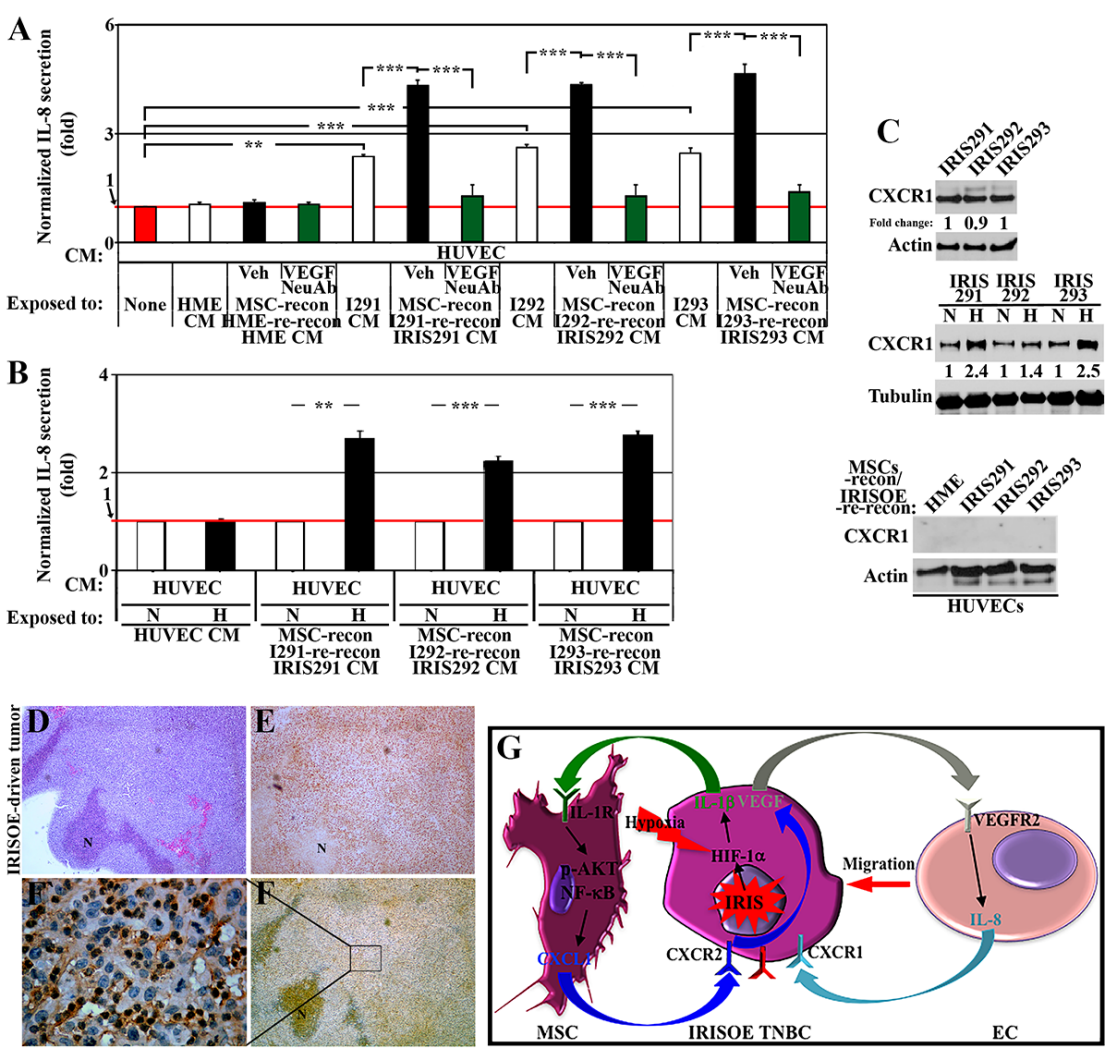
ECs respond by secreting IL-8 to re-activate IRISOE TNBC cells **(A)** Normalized IL-8 level detected by ELISA in CM from HUVECs unexposed (red bar), or exposed to HME, IRIS291, IRIS292 or IRIS293 CM (white bars), or same cells CM reconditioned by MSCs contact (24h) then reconditioned by the same mammary cell line contact (24h) in the absence (black bars) or presence of VEGF NeuAb (green bars). **(B)** Normalized IL-8 level detected by ELISA in the CM of HUVECs exposed to naïve HUVEC CM, or CM from normoxic (white bars), or hypoxic (24h, black bars) IRIS291, IRIS292 or IRIS293 cells reconditioned by MSC contact (24h). **(C)** The expression of CXCR1 on the surface of normoxic or hypoxic (24h) IRIS291, IRIS292 or IRIS293 cells (upper), or HUVECs exposed to CM from HME, IRIS291, IRIS292 or IRIS293 cells reconditioned by MSCs contact (24h), then by the same mammary cell line contact (24h, lower). **(D-F)** IHC analysis of CXCR1 expression in 1° IRISOE orthotopic tumor. (F`) Higher magnification image of the area squared in F. Scale bars: 500μm in D-F, and 50μm in F`. **(G)** Proposed model for signaling axis between IRISOE entrained by MSCs and HUVECs. Note, actin blots are the same as in Figure 5E, and that this experiment and the one in Figure 5E represent positive controls for one another.

ECs are a prominent source of support for aggressive tumor cells, in part by secreting factors with aggressiveness inducing abilities, such as IL-8 [45]. To investigate the role ECs play in enhancing IRISOE TNBC cells aggressiveness, normoxic or hypoxic (24h) naïve HME or IRISOE cells CM reconditioned by MSC contact (24h) then the same tumor cell line contact (24h) were added to naïve HUVEC (24h, for experimental details see Supplementary Figure 4). ELISA analysis revealed that naïve HUVECs secrete low-level IL-8 (taken as 1, red bar and line, Figure 6A). When exposed to naïve HME cells CM naïve HIVEC secrete low-level VEGF, while high-levels when exposed to IRIS291, IRIS292, or IRIS293 cells CM (white bars, Figure 6A). Moreover, IRIS291, IRIS292, or IRIS293 (not naïve HME) cells CM reconditioned by MSC contact then by the same tumor cell line contact promoted even further secretion of IL-8 from naïve HUVECs (compare black to white bars, Figure 6A). Including the VEGF NeuAb before exposing CM to naïve HUVECs blocked IL-8 secretion (compare green to black bars, Figure 6A). In addition, if the original CM was from hypoxic IRISOE tumor cells the secretion of IL-8 from HUVEC increased even further (Figure 6B). Together suggest that although IRISOE tumor cells normally secrete VEGF, entrainment by hypoxia and/or MSCs contact exacerbates the secretion leading to recruitment of ECs to the vicinity of IRISOE tumor cells, most likely into the aggressiveness niche, *in vivo*, and activating them to secrete IL-8. The data also suggest that VEGF action is unidirectional from IRISOE tumor cells to ECs.

CXCR1 is the preferred receptor for IL-8 [46]. Analyzing membrane fractions by Western blot showed that IRIS291, IRIS292, and IRIS293 tumor cells express high level CXCR1 on their surface (Figure 6C, upper), increased even further when cells exposed (24h) to hypoxia (Figure 6C, middle). In contrast, naïve HUVEC exposed (24h) to naïve HME, IRIS291, IRIS292, or IRIS293 cells CM reconditioned by MSCs contact (24h) then the same cell line contact (24h, see experimental details Supplementary Figure 4) show no CXCR1 expression on their surface (Figure 6C, lower). Accordingly, IHC staining showed that only tumor cells within orthotopic 1° IRISOE-driven mammary tumors express CXCR1 (Figure 6D-6F). Together suggest that IL-1β secreted by IRISOE tumor cells activates in paracrine fashion MSCs to secrete CXCL1 that also in paracrine fashion activates IRISOE tumor cells to secrete VEGF. VEGF in paracrine fashion triggers expression of VEGFR2 on the surface of naïve ECs, leading to their recruitment to the vicinity of IRISOE cells, most likely in the aggressiveness niche, *in vivo*, and their activation to secrete high levels of IL-8. Hypoxia and/or MSCs contact exacerbates all the events, even VEGFR2 expression on ECs and CXCR1 on IRISOE tumor cells (see Figure 6G).

### IRISOE TNBC cells activate the microenvironment, *in vitro*

**Figure 7:**
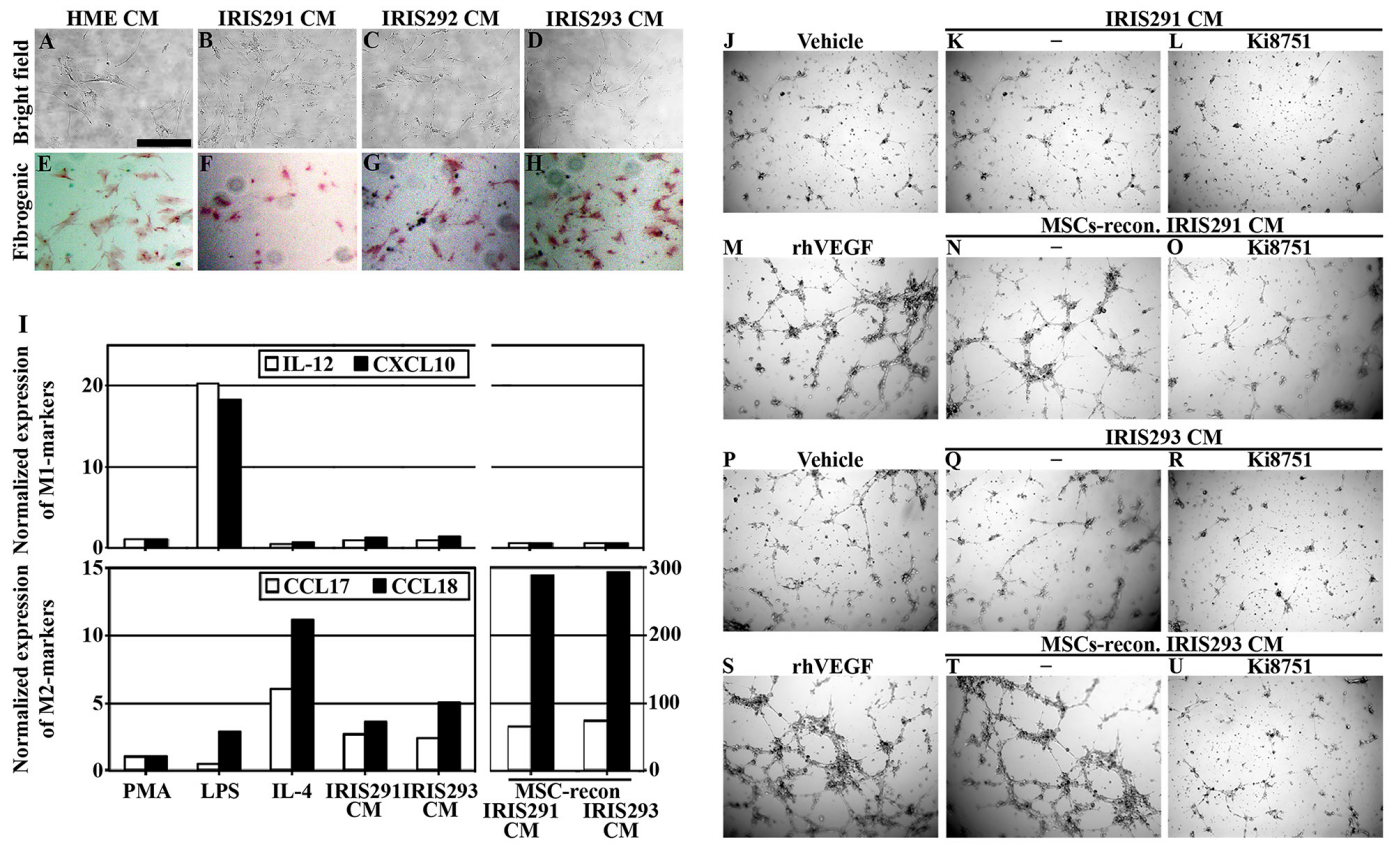
IRISOE TNBC tumor cells activate the microenvironment *in vitro* Bright field microscopy **(A-D)** or PicroSirius, fibrogenic staining **(E-H)** of MSCs exposed to HME, IRIS291, IRIS292 or IRIS293 cells CM, respectively. Scale bars: in (A-H) is 10μm. **(I)** Quantitative RT/PCR analysis of biomarkers for M1- (i.e. IL-12, and CXCL10, upper), or M2- (CCL17, and CCL18, lower) polarization of macrophage after exposure to; PMA, LPS, IL-4, IRIS291 cells CM, or IRIS293 cells CM reconditioned or not by MSC contact. The effect on HUVECs tube formation ability following treatment with vehicle **(J** and **P)**, rhVEGF **(M** and **S)**, IRIS291 cells CM in the absence **(K)** or presence **(L)** of Ki8751, IRIS293 cells CM in the absence **(Q)** or presence **(R)** of Ki8751, IRIS291 CM reconditioned with MSCs contact in the absence **(N)** or presence **(O)** of Ki8751, or IRIS293 CM reconditioned with MSCs contact in the absence **(T)** or presence **(U)** of Ki8751.

Naïve MSCs exposed to naïve HME CM for 7 days (medium changed daily) maintained their naïve MSCs morphology of large nuclei and cytoplasms (Figure 7A and Supplementary Figure 7A). They also maintained their ability to equally adopt adipogenic (Oil-Red O staining, Supplementary Figure 7E and Supplementary Figure 7M), osteogenic (Alizarin staining, Supplementary Figure 7I and Supplementary Figure 7N), chondrogenic (Alcian blue staining, not shown Supplementary Figure 7O) and fibrogenic (PicroSirius staining, Figure 7E and Supplementary Figure 7P) fates. In contrast, MSCs exposed to IRIS291, IRIS292, and IRIS293 CM for 7 days (media changed daily) showed more elongating and fibroblastic morphology (compare Figure 7B-7D to A and Supplementary Figure 7B-7D to A), and greater tendency to adapt the fibrogenic fate (Figure 7F-7H and Supplementary Figure 7P) on the expense of the adipogenic (Supplementary Figure 7F-7H and Supplementary Figure 7M), osteogenic (Supplementary Figure 7J-7L and Supplementary Figure 7N) and chondrogenic (not shown and Supplementary Figure 7O) fates. Together suggest that IRISOE TNBC cells secretome skews MSCs differentiation towards the aggressiveness-promoting CAF fate, *in vitro*.

THP1-macrophages exposure (24h) to lipopolysaccharide (LPS) polarizes them towards anti-tumor M1-macrophages [39], as detected by the increased expression of the M1-macrophage biomarkers; IL-12 and CXCL10 in them (see RT/PCR analysis, Figure 7I, upper). In contrast, exposure to IL-4 and/or IL13 (24h) polarizes them towards pro-tumor M2-macrophage fate (*aka* tumor associated macrophages or TAMs) [39], as detected by increased expression of the M2-macrophage biomarkers; CCL17 and CCL18 in them (see RT/PCR analysis, Figure 7I lower). Moreover, unlike luminal A/ER^+^-tumors that support anti-tumor M1-polarization, TNBC tumors support pro-tumor M2-polarization [24, 25].

IRIS291, and IRIS293 cells CM (i.e. contains low-level CCL2, see Figure 3A and 3C) induced expression of CCL17 and CCL18 (Figure 7I, lower) not IL-12 and CXCL10 (Figure 7I, upper) in THP1-macrophages. Furthermore, exposure to IRIS291 or IRIS293 CM reconditioned by MSC contact (24h, i.e. contains high-level CCL2, see Figure 3C) induced an additional 20 and 60 fold higher increase in the level of CCL17 and CCL18 in THP1-macrophages (compare far right to last bars on the left, Figure 7I, lower). Together suggest that MSC contact entrains IRISOE cells secretome to polarize macrophages towards the aggressiveness-promoting TAM cells polarization, *in vitro*.

Finally, HUVEC layered in matrigel-coated wells were incubated with EC-medium containing a vehicle, rhVEGF, concentrated IRIS291, or IRIS293 CM reconditioned or not by MSCs or in the absence or presence of the VEGFR2 specific inhibitor; Ki8751 [47]. As expected, 6h later no/few very small tubes formed in a vehicle containing cultures (Figure 7J and 7P), while elaborate tube formation in rhVEGF-supplemented cultures was observed (Figure 7M and 7S). Without MSC contact, IRIS291 CM (Figure 7K) and IRIS293 CM (Figure 7Q) promoted marginal tube formation, whereas elaborate and extensive tube formation was detected when IRIS291 CM (Figure 7N) or IRIS293 CM (Figure 7T) was re-conditioned by MSC contact. This tube formation was very sensitive to Ki8751 (compare Figure 7O to 7N for IRIS291 and Figure 7U to 7T for IRIS293). Together suggest that MSCs contact entrains IRISOE cells secretome to promote neo-angiogenesis, *in vitro*.

### IRISOE TNBC cells activate the microenvironment, *in vivo*

**Figure 8:**
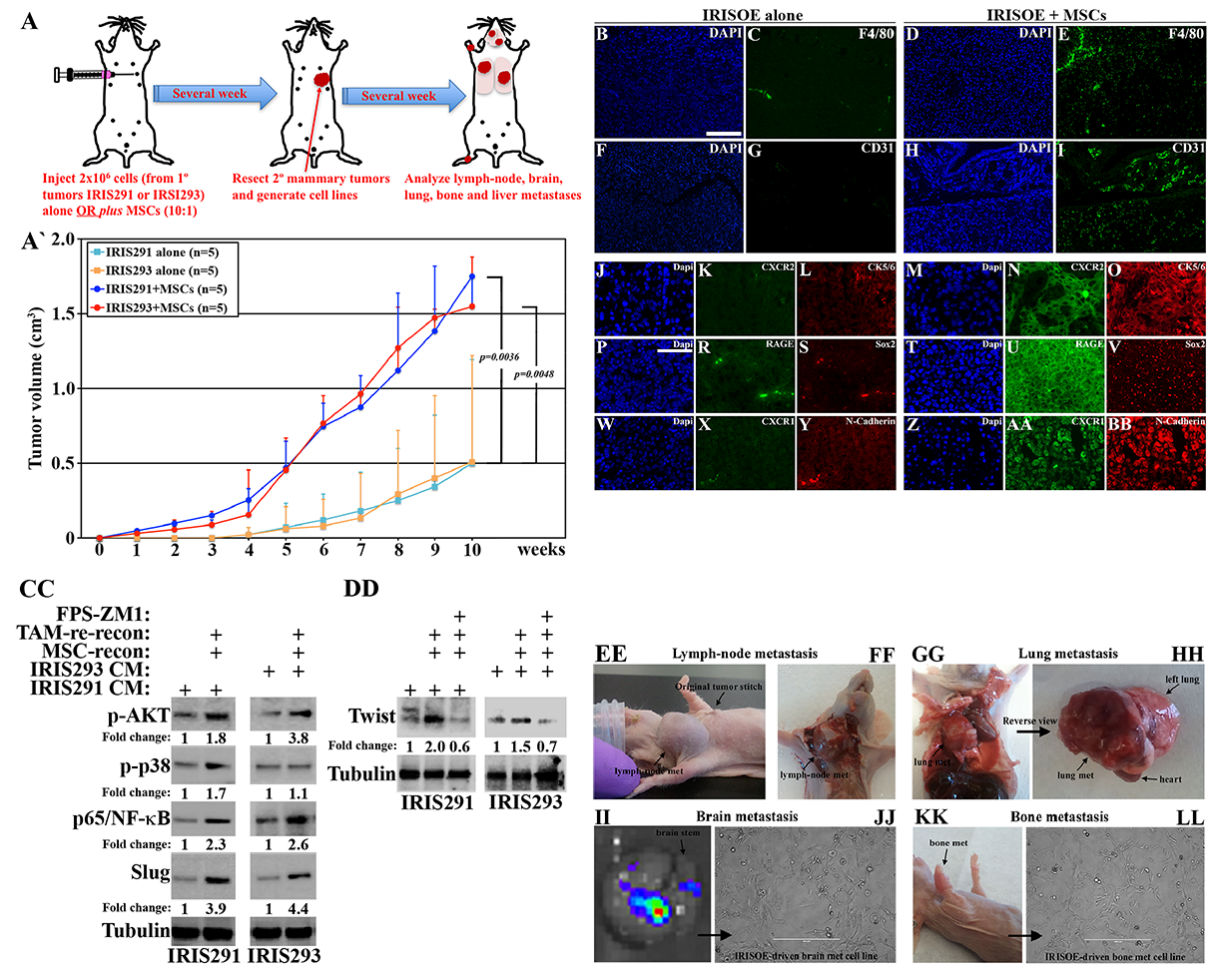
IRISOE TNBC tumor cells activate the microenvironment, *in vivo* **(A)** Schematic representation of the experiments performed. (A`) The volume of orthotopic mammary tumors developed in Nu/Nu mice injected with 2×10^6^ IRIS291 or IRIS293 cells alone or admixed with 10% MSCs (n=5/each). F4/80^+^ cells in IRISOE-driven tumor developed in the absence **(B, C)** or presence of **(D, E)** of MSCs (admixed 10:1). CD31^+^ cells in IRISOE-driven tumor developed in the absence **(F, G)** or presence of **(H, I)** of MSCs (admixed 10:1). Co-localization of CXCR2/CK5 cells in IRISOE-driven tumor developed in the absence **(J-L)** or presence of **(M-O)** of MSCs (10:1). Co-localization of RAGE/Sox2 cells in IRISOE-driven tumor developed in the absence **(P-S)** or presence of **(T-V)** of MSCs (10:1). Co-localization of CXCR1/CDH2 cells in IRISOE-driven tumor developed in the absence **(W-Y)** or presence of **(Z-BB)** of MSCs (10:1). Scale bars: 100μm in B-I, and 50μm in J-BB. (CC) Activated AKT, p38, NF-κB/p65, slug levels in IRISOE cells exposed to IRIS291 or IRIS293 cells CM, reconditioned with MSCs contact (24h), then re-reconditioned with the same cell line contact (24h), then re-reconditioned with THP1-macrophages contact (24h). (DD) Expression of Twist in IRISOE cells exposed to IRIS291 or IRIS293 cells CM, reconditioned with MSCs contact (24h), then with the same cell line contact (24h), then with THP1-macrophage contact (24h) in the absence or presence of the RAGE inhibitor FPS-ZM1. (EE and FF) Lymph-node, (GG and HH) lung, (II and JJ) brain, and (KK and LL) bone metastasis appeared in mice injected with IRISOE cells:MSC (10:1).

We injected 2×10^6^ IRIS291 or IRIS293 cells alone or admixed with 2×10^5^ MSC in female Nu/Nu mice mammary fat pads (n=5 per cell line). At 10 weeks (the allowed time-point), only 2 IRIS291 cells (average 501 ± 692 mm^3^) and 2 IRIS293 cells (average 510 ± 708 mm^3^) injected mice developed tumors (Figure 8A and A`). In contrast, all 5 mice injected with IRIS291 + MSCs (average 1750 ± 130 mm^3^) and IRIS293 + MSCs (average 1549 ± 212 mm^3^) developed tumors (Figure 8A and A`). Tumors developed using IRISOE cells + MSC contained higher levels of F4/80^+^-TAMs (compare Figure 8D and 8E to 8B and 8C) and CD31^+^-ECs (compare Figure 8H and 8I to 8F and 8G) compared to those developed using IRISOE cells alone.

Tumors developed in the presence MSCs also showed increased number of CXCR2 (compare Figure 8M and 8N to 8J and 8K)/CK5/6 (basal-biomarker, compare Figure 8O to 8L) co-expressing cells, increased number of RAGE (compare Figure 8T and 8U to 8P and 8R)/Sox2 (stemness-enforcer, compare Figure 8V to 8S) co-expressing cells, and increased number of CXCR1 (compare Figure 8Z and 8AA to 8W and 8X)/N-Cadherin (in EMT-inducer, compare Figure 8BB to 8Y) co-expressing cells. Together suggest that MSCs entrain aggressiveness, including enhanced infiltration of TAMs and ECs, and enhanced basal, EMT and stem-like phenotypes in IRISOE tumor cells, *in vivo*.

To measure this experimentally, IRIS291 or IRIS293 cells CM (24h) reconditioned by MSCs contact (24h), then reconditioned by the same cell line contact (24h), then reconditioned by THP1-macrophages contact (24h) was added to the same cell line (24h) in the presence or absence of the RAGE-specific inhibitor; FPS-ZM1 [48] (for experimental details see Figure 8CC and Supplementary Figure 4). Examining total protein extracts using Western blot showed enhanced expression of activated AKT, ERK, p65/NF-κB, slug (Figure 8CC), and Twist (compare middle to left lanes, Figure 8DD) in the absence of PFS-ZM1. FPS-ZM1 blocked the upregulation in Twist expression in IRIS291 or IRIS293 under the same conditions (compare right to middle lanes, Figure 8DD). Together suggest that cross talk between IRISOE cells, MSC and TAM within the aggressiveness niche, *in vivo* culminates on generating more aggressive IRISOE tumor.

To experimentally investigate that, we resected the tumors generated above and allowed the mice to live until metastasis appeared. Within 2 months all mice originally injected with IRISOE cells + MSC developed either lymph nodes (Figure 8EE and 8FF), or distant metastasis, e.g., lung (Figure 8GG and 8HH), brain (Figure 8II and 8JJ), or bone (Figure 8KK and 8LL). Noteworthy, mice developed tumors following IRISOE alone cells injection did develop metastasis to lung and bones, however, at much later time-point, which could be due to the delayed recruitment of mouse MSC and the formation of the proposed “aggressiveness niche”. We concluded that even *in vivo*, injecting IRISOE tumor cells with MSCs gives them an advantage for quickly and efficiently recruit TAMs and ECs, which enhances tumor cells aggressiveness, including early dissemination and metastasis.

### The efficacy of inhibiting IRISOE TNBC cells secretome, *in vivo*

**Figure 9:**
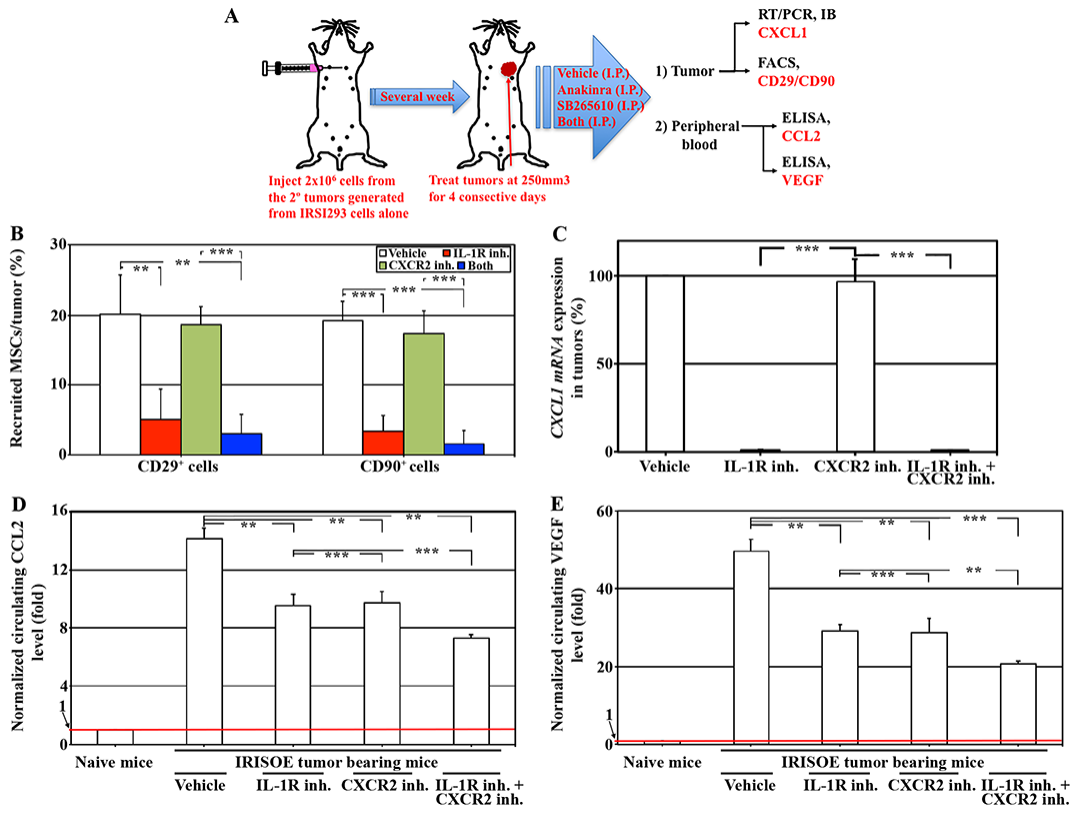
The efficacy of inhibiting IRISOE TNBC cells secretome, *in vivo* **(A)** Schematic representative of experiments performed. **(B)** FACS analysis for CD29 and CD90 (MSC specific biomarkers), and **(C)** qRT-PCR analysis for *CXCL1 mRNA* expression in 3° IRISOE tumors developed in Nu/Nu mice treated with vehicle, Anakinra, SB265610, or both. Circulating CCL2 **(D)** or VEGF **(E)** levels in PB from naïve Nu/Nu mice or mice bearing 3° IRISOE orthotopic mammary tumor and treated for 4 days with vehicle, Anakinra, SB265610, or both.

To elucidate the clinical benefit of inhibiting the proposed “aggressiveness niche” in breast cancer patients, the secondary (2°) tumors developed above were used to generate cells lines. One such cell line developed in the absence of MSCs was implanted (instead of 1° tumor cell line to expedite tumor formation) in 20 female Nu/Nu mice mammary fat pads (2×10^6^ cells/mouse). At a ∼250mm^3^ tumor volume, mice were divided into 4 groups intraperitoneally injected with: vehicle, Anakinra (human grade IL-1β antagonist; IL-1ra, 10mg/kg [49]), SB265610 (CXCR2 inhibitor, 2mg/kg [14]) or both (same concentrations) for 4 consecutive days. On day 5, tumors and peripheral blood (PB) from all mice were collected. Tumors were digested into single cell suspension, and sera were isolated from the PB see Figure 9A.

FACS analysis with two mouse MSC specific biomarkers; CD29 (expressed by MSC from bone marrow, synovium, and epiphysis origin [50]), and CD90 (expressed by adults mouse MSCs from adipose tissue origin [51]) showed that while vehicle treated IRISOE orthotopic mammary tumor contain ∼20% MSC (white bar, Figure 9B), Anakinra (compare red to white bars, Figure 9B), not SB265610 (compare green to white bars, Figure 9B) decreased that to ∼5%. No further decrease was detected in the combinatorial treatment (compare blue and red bars, Figure 9B). Moreover, real-time qRT/PCR and Western blot showed a significant decrease in *CXCL1 mRNA* (Figure 9C) and protein (Supplementary Figure 8) in Anakinra, not SB265610 treated IRISOE tumors. Together suggest that inhibiting IL-1β signaling significantly decrease recruitment of mouse MSCs into IRISOE TNBC tumors, and their activation to produce and secrete CXCL1, *in vivo*.

Furthermore, compared to naïve mice, IRISOE tumors-bearing mice showed much higher levels of circulating CCL2 (Figure 9D) and VEGF (Figure 9E) according to ELISA analysis performed on sera isolated from PB of these mice. Inhibiting IL1R or CXCR2 signaling in mice significantly reduced the levels of circulating CCL2 (Figure 9D) and VEGF (Figure 9E), and inhibiting signaling of IL-1R *plus* CXCR2 simultaneously had an additive effect (Figure 9D and 9E). Together suggest that IL-1β secreted by IRISOE tumor cells recruits MSC into tumors’ aggressiveness niche, activates them to secrete CXCL1, which entrains IRISOE tumor cells to secrete higher local and systemic levels of CCL2 and VEGF, *in vivo*.

### High level IL1β *plus* CXCL1 *plus* CCL2 *plus* S100A8 *plus* VEGF *plus* IL8 correlates with adverse outcomes in breast cancer patients

**Figure 10:**
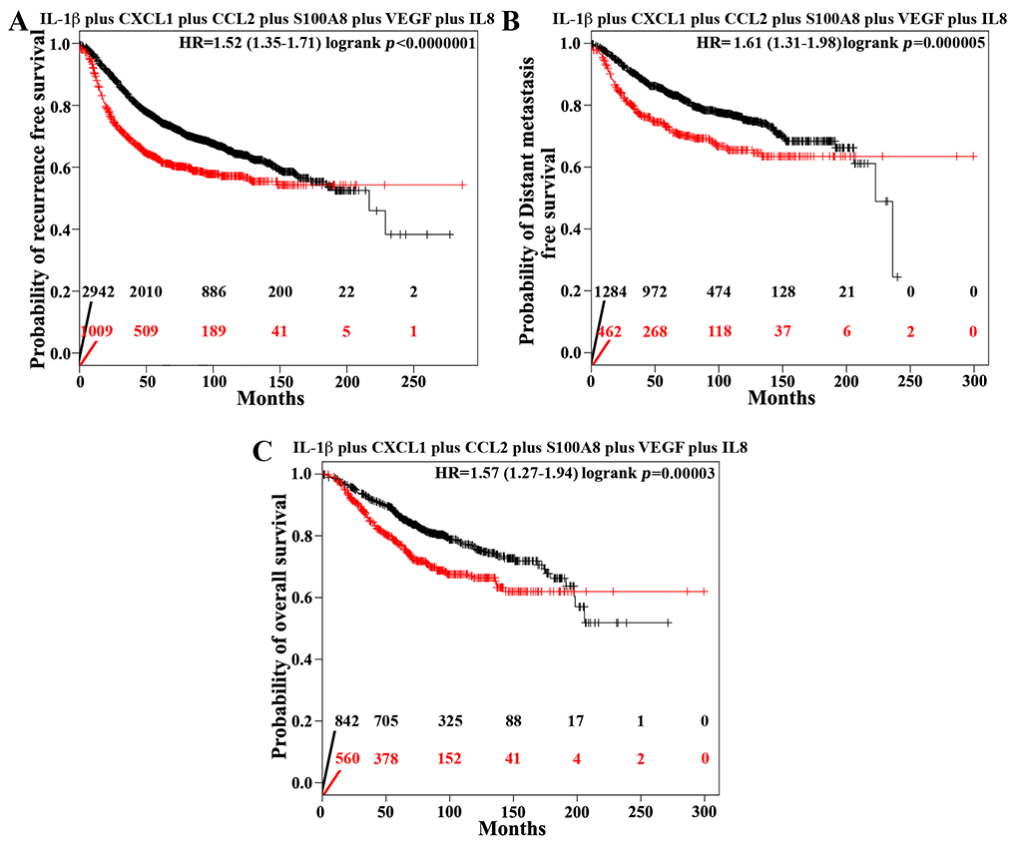
High levels IL1β *plus* CXCL1 *plus* CCL2 *plus* S100A *plus* VEGF plus IL-8 correlates with adverse outcomes in breast cancer patients Kaplan Meier analysis of recurrence free survival **(A)**, (n=3951), distant metastasis free survival **(B)**, (n=1746), or overall survival **(C)**, (n=1402) in IL1β *plus* CXCL1 *plus* CCL2 *plus* S100A *plus* VEGF plus IL-8 low expressing (black lines and numbers) vs. high expressing (red lines and numbers) breast cancer patients.

The data so far suggest that high-level IL-1β secreted by IRISOE TNBC cells activates MSCs to secrete CXCL1 to entrain IRISOE TNBC cells to secrete high-levels CCL2 to activate TAMs to secrete S100A8, and VEGF to activate ECs to secrete IL8, which culminates on formation of aggressive IRISOE TNBC mammary tumors. To evaluate the high expression IL-1β, CXCL1, CCL2, S100A8, VEGF and IL8 as surrogate biomarker for aggressive breast cancers (e.g., IRISOE TNBC tumors), meta-analysis based biomarker assessment using the online tool Kaplan Meier Plotter (http://kmplot.com/analysis [52]) was performed. We evaluated the association between the expression of IL-1β *plus* CXCL1 *plus* CCL2 *plus* S100A8 *plus* VEGF *plus* IL8 and outcomes in several cohorts of breast cancer patients. Normalized expression levels of IL1-β, CXCL1, CCL2, S100A8, VEGF, and IL8 are available for every patient in each cohort; the individual expression levels were summed, and each cohort was then dichotomized into patients with high or low expression of IL1-β, CXCL1, CCL2, S100A8, VEGF, and IL8 using the median of the summed expression levels in each cohort as the split point. Subsequently, Kaplan-Meier plots and logrank statistics were calculated to compare the subgroups with high or low expression. This analysis revealed that patients with high-level IL-1β *plus* CXCL1 *plus* CCL2 *plus* S100A8 *plus* VEGF *plus* IL8 show significantly reduced recurrence free survival (RFS, *p<0.023476R21*, Figure 10A), significantly reduced distant metastasis free survival (DMFS, *p=0.000005*, Figure 10B), and significantly reduced overall survival (OS, *p=0.00003*, Figure 10C) compared to patients with low-level IL-1β *plus* CXCL1 *plus* CCL2 *plus* S100A8 *plus* VEGF *plus* IL8. Median RFS was 60 vs. 26 months, median DMFS was 126 vs. 48 months, and median OS was 126 vs. 66 months for patients with low-level vs. high-level expression of the six cytokines. Together, suggest that high local or systemic IL-1β *plus* CXCL1 *plus* CCL2 *plus* S100A8 *plus* VEGF *plus* IL8 in breast cancer patients represents a diagnostic biomarker for the existence of IRISOE TNBC tumor, and propensity to reduced RFS, DMFS, or OS. This implies treating these patients with an IRIS blocker could interrupt tumor cells-MSCs/TAMs/ECs cross-talks leading to better patients’ outcomes. Additionally, if IRISOE TNBC tumors are diagnosed at an earlier time point (i.e. early lesion) by this diagnostic test, the treatment might potentially be curative.

## DISCUSSION

IRISOE induces triple negative phenotype in human [53], and pre-clinical orthotopic mouse model [29, 31] breast cancers. Indeed, cells overexpressing IRIS show many of the properties of TNBC cells, including high expression of the basal-biomarkers, EMT-inducers, and stemness-enforcers, as well as low expression of the BRCA1 protein, in culture [30, 31], orthotopic mammary tumors [29, 30], as well as human tumor samples [53]. Like human TNBC tumors, IRISOE-induced orthotopic mammary tumors show a large central acellular core of necrosis surrounded by hypoxic area [29]. Recently, it was proposed that this core indicates a higher risk for metastasis and mortality in TNBC patients [54, 55]. We recently proposed to name this core “the aggressiveness niche [35]”, where metastatic precursors develop. How can a death (i.e. necrotic) core be involved in the development of metastatic precursors? It is possible that cells that overcome the unfavorable conditions within the primary tumors’ core, including detachment from the ECM, attack by immune cells, hypoxia, growth factor-deprived environment, and increased cellular oxidative stress (i.e. ROS production and DNA damage) could develop greatly improved fitness to survive these stressful conditions, disseminate and become metastatic precursors [56].

Our previous analysis of IRISOE-induced vs. Ras^V12^OE-induced orthotopic mammary tumors [29] revealed that only IRISOE-induced orthotopic tumors contained this necrotic/hypoxic/inflamed cores. Despite that the kinetic of both tumors growth was identical. This suggested that IRISOE-driven tumors only are rapidly growing tumors, so much that they compensate for the loss of cells by necrosis with newly generated cells to maintain similar tumor size as the non-necrotic/slow growing Ras^V12^-driven tumors [29].

In line with these interpretations, we also found that unlike the Ras^V12^OE-driven tumors that are circumscribed, non-invasive low-grade tumors with excessive glandular structures, and epithelial biomarkers, ERα, and BRCA1 protein expression indicating their luminal phenotype, IRISOE-driven tumors are invasive high-grade tumors with prominent spindle cell component, and EMT-inducers but no ERα or BRCA1 proteins expression indicating their TNBC phenotype [29]. Together, suggest that IRISOE-driven tumors microenvironment, especially, the necrotic/hypoxic/inflamed core (i.e., aggressiveness niche), and the selective bi-directional interactions between tumor cells and the non-transformed microenvironment entities within this core produce IRISOE TNBC metastatic precursors [35]. Intra-tumor necrosis, chronic inflammation, and hypoxia within aggressive breast cancers, such as TNBCs are a well-known independent prognostic factor for low RFS [57].

We propose that aggressiveness niche micro-environment promotes the formation of metastatic precursors by enhancing expression of pro-metastatic genes in these, such as tenascin-C, and matrix metalloproteinases [58]. The most prominent transcription factor involved in activating expression of such genes is NF-κB [27, 59]. However, some studies estimate that only 60% of the pro-metastatic genes [58] are direct transcriptional targets for NF-κB, which suggests an involvement of other transcriptional factors. This implies that additional transcriptional signaling, such as those we identified here, e.g., HIF-1α, AKT, and ERK could be also involved in activating the remaining 40% of the pro-metastatic genes within the proposed aggressiveness core. It is thus possible to predict that combining cytotoxic chemotherapy with IRIS (in progress), NF-κB and/or HIF-1α inhibitors could improve the outcomes for women with IRISOE TNBC tumors. How would such patients be identified? According to the current study, patients would qualify for such a treatment if they show within their tumors, or systemically enhanced levels of IL-1β *plus* CXCL1 *plus* CCL2 *plus* S100A8 *plus* VEGF *plus* IL-8. This would be a very good example of personalized treatment option for IRISOE TNBC patients.

Increasing evidence suggest chemokines are essential mediators of the dialog between tumor cells and their microenvironment by activating NF-κB-dependent transcription [60]. The fact that aggressive IRISOE TNBC cells produce and secrete high-level IL-1β in HIF-1α and NF-κB-dependent manner [61] is consistent with recent reports showing IL-1β is a critical mediator of inflammation leading to tumor progression within breast tumor microenvironment [62, 63]. Like IRIS [33], the expression of IL-1β is steadily increase from very low in normal breast epithelium, to relatively high in patients with ductal carcinoma in situ (DCIS), to even higher in patients with invasive ductal carcinoma (IDC) with no relapse, and is highest in patients with IDC with relapse [64]. CXCL1 overexpression primes breast cancer cells for survival in metastatic sites [65]. Additionally, other chemokines produced by MSCs, TAMs, and ECs [66] within the IRISOE TNBC aggressiveness niche could enhance tumor growth, promote tumor cells invasion or metastatic capabilities [67-69], in part as we elucidated here by increasing the number of cancer stem cells within that niche [70], and facilitating trans-endothelial migration through production of e.g., VEGF/VEGFR2, or other related mechanisms [71].

It is interesting that while IL-1β, CCL2, and VEGF are secreted at high levels from hypoxic and/or MSCs-entrained IRISOE TNBC tumor cells, their receptors, IL-1R, CCR2, and VEGFR2 were only observed on MSCs, TAMs, and ECs, respectively. This implies a paracrine interaction from tumor cells to the microenvironment. On the other hand, CXCR2, RAGE, and CXCR1 the receptors of CXCL1, S100A8 and IL-8 secreted in response to activation by IRISOE TNBC cells from MSCs, TAMs, and EC, respectively are only expressed on IRISOE TNBC tumor cells also raises the intriguing possibility that a reciprocal paracrine interaction from the microenvironment to IRISOE TNBC tumor cells also exist. It is confirmed from the data presented here that these factors activate aggressiveness within IRISOE TNBC cells within the niche by, for instance activating AKT, MAPK, and NF-κB in IRISOE TNBC tumor cells. If correct, this implies an intrinsic aggressiveness-inducing ability in IRISOE TNBC tumor cells [35] by activating the microenvironment, and a reciprocal extrinsic aggressiveness-inducing ability by the microenvironment by activating IRISOE TNBC metastatic potential.

Our data raise another interesting possibility. Since the IRISOE TNBC secretome is intrinsic it would be secreted by cells within the core as well as at the tumor’s periphery. How then metastatic precursors are produced within the aggressiveness niche? It is possible that as we showed the secretome level is enhanced by the microenvironment such as hypoxia and inflammation in the aggressiveness niche. This could generate a gradient to which more culprit cells; MSCs, TAMs, and ECs are recruited and thus more (or stronger) bi-directional interactions between tumor cells-microenvironment cells are formed and thus the generation of metastatic precursors within that niche only.

**Figure 11:**
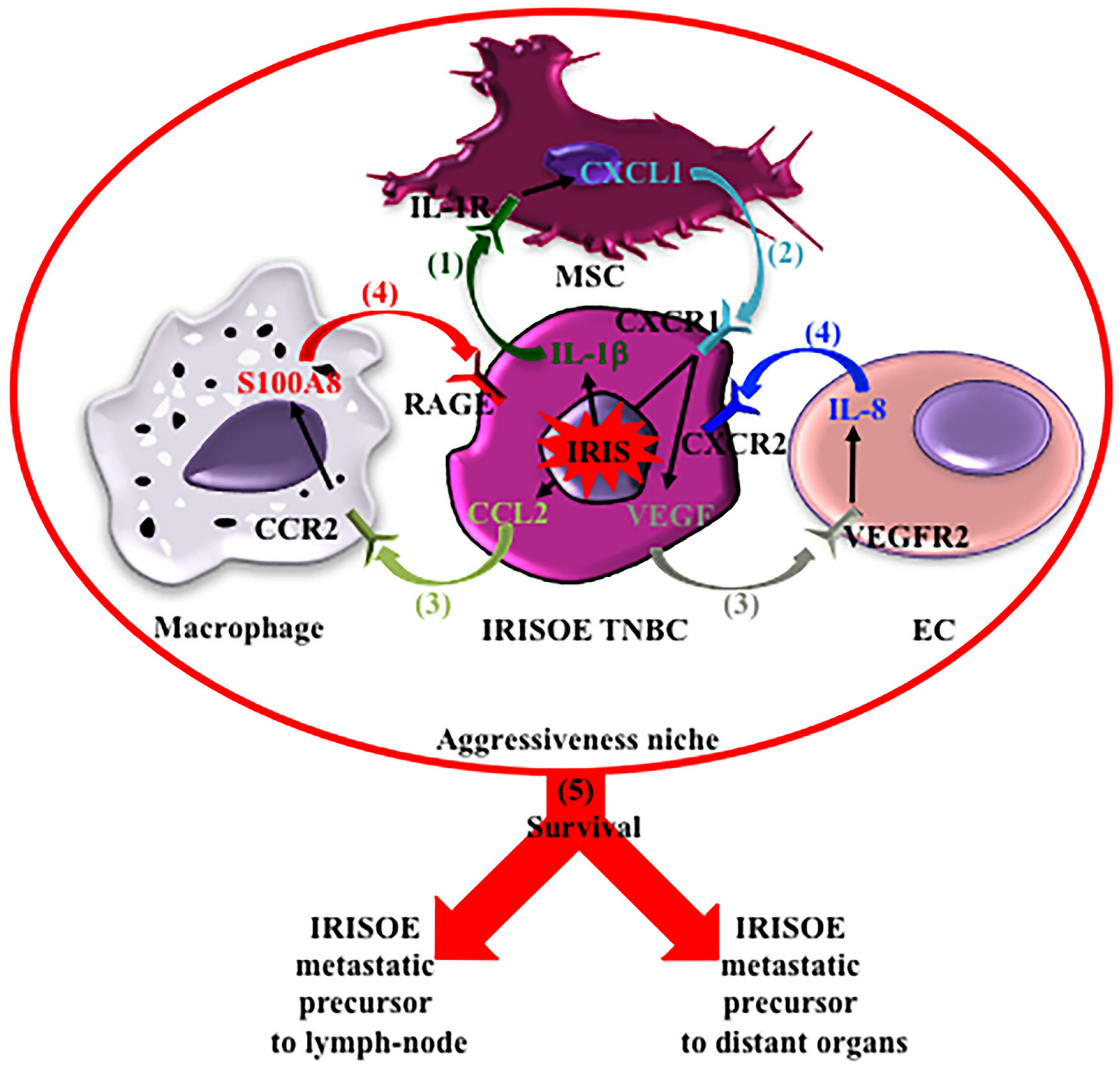
Proposed model for the aggressiveness niche hypothesis and the contribution of the bi-directional interactions described herein in the production of IRISOE TNBC metastasis precursors

Our working hypothesis (Figure 11) is that within the aggressiveness niche, IL-1β secreted by metabolically stressed, hypoxic and inflamed IRISOE TNBC tumor cells recruits MSCs to the niche (step 1, Figure 11), and activates them to secrete CXCL1 (step 2, Figure 11). CXCL1 functions to entrain IRISOE TNBC tumor cells to secrete higher levels of CCL2 and VEGF (steps 3, Figure 11). CCL2 recruits TAMs to the niche and activates them to secrete S100A8/9, while VEGF recruits ECs to the niche and activates them to secrete IL-8 (steps 4, Figure 11). In concert with CXCL1, S100A8/9 and IL-8 entrain IRISOE tumor cells to survive the harsh conditions within the niche (step 5, Figure 11) to become metastatic precursors (Figure 11). This network of bi-directional interactions provides a mechanistic explanation for the elevated aggressiveness traits of IRISOE TNBC cells co-injected with MSCs, and a mechanism linking chemo-resistance and metastasis of IRISOE TNBC cells, with opportunities for intervention. For instance, blockers for the receptors described herein expressed on IRISOE TNBC cells’ or the stromal cells’ surface, e.g., the FDA approved “Anakinra” could break these bi-directional interactions and augment the efficacy of chemotherapy against IRISOE TNBCs and particularly against metastasis.

We recently showed that IRISOE TNBC metastatic precursors disseminate from early disease lesions [34]. Accordingly, it is possible that because of the abundant hypoxia and inflammation facing the earliest normal mammary cells that transformed into IRISOE TNBC cells that these cells disseminate as IRISOE TNBC metastatic precursors. It is thus possible to suggest that treatment of IRISOE TNBC metastatic cells should start at the early disease lesion stage, using diagnostic tools such as those proposed above.

## MATERIALS AND METHODS

### Cell culture

The doxycycline (Dox)-inducible IRISOE cell lines (IRISOE1-5) generation and maintenance was described earlier [28]. These cell lines develop into orthotopic IRISOE mammary tumors when injected in SCID mice and the mice given Dox-supplemented drinking water only (naïve HME don’t survive *in vivo* [29, 72]). Three cell lines “IRIS291, IRIS292, and IRIS293” were developed from these resected 1° orthotopic IRISOE tumors and were maintained in Dox-supplemented RPMI 1640 medium containing 10% fetal bovine serum (FBS). Human bone marrow-derived MSC isolated from volunteers, verified, and propagated by Texas A&M (HSC COM Institute for Regenerative Medicine). In our laboratory MSC were maintained in MEM/α- GlutaMAX medium supplemented with 17% FBS. HUVECs were obtained from American Type Culture Collection (ATCC), and were maintained in MCDB 131 medium supplemented with 7.5% FBS, 2mM GlutaMAX, 20mM HEPES, 10ng/ml epidermal growth factor, 1ng/ml bovine fibroblast growth factor and 1μg/ml hydrocortisone. Primary monocytes (THP-1) cell line was obtained from ATCC, and was maintained in RPMI 1640 medium supplemented with 10% FBS. To differentiate THP-1 cells into un-polarized macrophages, cells were exposed to 200nM phorbol 12-myristate 13-acetate (PMA, Sigma, St Louis, MO, USA) for 4 days. Macrophages polarization into M1-macrophages was accomplished by incubation with 100ng/ml of lipopolysaccharide (LPS, Sigma), and into M2 by incubation with 20ng/ml of interleukine-4 (IL-4, R&D Systems, Minneapolis, MN, USA) for 24h in accordance with previous protocol [73]. All commercial cell lines were authenticated by STR profiling and tested for mycoplasma contamination.

### Antibodies

See list in Supplementary Table 1.

### Cytokine array

IRISOE1-5 grown in the absence of Dox (i.e. naïve HME) and in the presence of Dox (i.e. IRISOE1-5 or HME/IRIS) cells were assessed for differential factors. Briefly, conditioned media (CM) from equal number of either cell line plated in serum-free medium for 20h under standard conditions was used to screen cytokine, chemokine and growth factors antibody arrays (RayBio, Norcross, GA, USA) performed according to the manufacturer’s instructions and as described previously [74].

### siRNA transfection

Naïve HME, IRIS291, and IRIS293 cells were seeded at a density of 3×10^5^ cells/well in a 6-well plate. After 16-18h, transient transfection of siLuc and siHIF1α siRNA (see Supplementary Table 2) was carried out using Xfect™ Transfection reagent (Clonetech Laboratories, Inc., Mountain View, CA, USA) according to the manufacturer’s instructions. After 48h media was changed and cells were exposed to normoxia (20% O_2_) or hypoxia (1% O_2_) for an additional 24h, when CM were collected for ELISA analysis.

### Conditioned media transfer experiment

The protocol is schematically presented in Supplementary Figure 4. Briefly, normoxic (20% O_2_ for 24h) or hypoxic (1% O_2_ for 24h) naïve HME, IRIS291, IRIS292, or IRIS293 CM was directly analyzed for secreted factors, and cells for surface receptors expression. Mammary cells CM (24h), reconditioned by MSC contact (24h) was analyzed for secreted factors, and MSC for surface receptors expression. Mammary cells CM (24h), reconditioned by MSC contact (24h), then reconditioned by same mammary cell line contact (24h) was analyzed for secreted factors and mammary cells surface receptors expression. Mammary cells CM, reconditioned by MSC contact (24h), then reconditioned by mammary cell contact (24h) then THP1-macrophages or HUVEC contact (24h) was analyzed for secreted factors, and THP1-macrophages or HUVEC for receptors expression. Finally, mammary cells CM (24h), reconditioned by MSC contact (24h), then reconditioned by mammary cell contact (24h), then reconditioned by THP1-macrophaes or HUVEC contact (24h), then finally reconditioned by the same mammary cell line contact (24h) was analyzed for secreted cytokines and mammary cells for surface receptors expression. At all steps, equal numbers of each cell type were seeded to avoid discrepancies due to cell number variations. At various steps in this protocol specific NeuAb or inhibitor was added. Secretion was investigated by ELISA surface receptors expression by Western blotting.

### Cytokine ELISA

Co-cultures CM or mice sera diluted in carbonate coating buffer (pH 9.6) were used to coat 96-well ELISA plates overnight at 4°C. Plates were then washed thrice with PBST (phosphate buffered saline- 0.05% Tween-20) and blocked with 2% bovine serum albumin for 1h at room temperature (RT). Plates were then incubated with primary antibody diluted in blocking solution for 2h at RT followed by HRP-conjugated secondary antibody for 1h at RT. Reaction was read using Western Lightning Plus-ECL (PerkinElmer, Waltham, MA, USA) as a substrate. Experiments were done in triplicates performed 3 separate times and shown as mean ± SD.

### Co-culture experiment

Boyden chambers (BD biosciences) of 8μm pore size (for migration) or 0.4μm-pore size (for secretome) analysis was used. Certain cells were layered in lower chamber with or without neutralizing antibodies and test cells were layered in the transwell inserts. Cells migrated to the lower compartment of Boyden chamber were counted and plotted. Occasionally, hypoxia for 24h was introduced.

### Western blot

Performed as previously described [74]. Briefly, protein lysates were prepared from membrane fraction or whole cell extracts by sonication in PBS containing protease and phosphatase inhibitor tablets (Thermo Scientific, Waltham, MA, USA) according to the manufacturer’s instructions. Protein concentration was estimated using Pierce™ BCA protein assay kit (Thermo Scientific, Waltham, MA, USA). Cell lysates were denatured in NuPAGE LDS sample buffer (Thermo Scientific) and were resolved on NuPAGE gels (Thermo Scientific) and electro-transferred to PVDF membrane. Membrane was blocked with 5% dry milk for 1h, washed thrice with PBST and subsequently incubated with primary antibody overnight at 4°C. Next day, blots were washed thrice with PBST and incubated with HRP-conjugated secondary antibody for 1h at RT, washed and developed using Western Lightning Plus-ECL as a substrate. Tubulin and actin were used as an internal loading control.

### Immunohistochemistry

Immunohistochemical analysis was performed as previously described [74] on 5μm thick paraffin-embedded sections of tumor tissue excised from IRISOE orthotopic mammary tumor generated in Nu/Nu mice. Briefly, sections were deparaffinized, rehydrated and washed in PBS. Antigen retrieval for IRIS staining was performed by incubating the slides in pepsin (10μM) for 20min at 37°C. Antigen retrieval for all other antigens was performed by boiling the slides in citrate buffer (pH 6.0) for 10min in the microwave. Slides were then cooled to RT and washed 3 times with PBS for 15min each. Slides were incubated in 3% hydrogen peroxide (H_2_O_2_) for 10min to block endogenous peroxidase activity unless fluorescence analyses were performed. After washing, slides were blocked with 10% normal goat serum for 1h at RT, washed and subsequently probed with primary antibodies overnight at 4°C in a moist chamber. After three PBS washes slides were incubated with horseradish peroxidase (HRP), or Alexa Fluor (488, 532, 568, or 647) conjugated secondary antibody for 1h at RT (depending on the analysis) and were washed with PBS. Slides that were stained with HRP-conjugated secondary antibody were developed with Vector DAB substrate kit (Vector laboratories, Burlingame, CA, USA) and counterstained with Meyer’s hematoxylin (Thermo Scientific) for 2min, washed, dehydrated and mounted with Permount (Thermo Fisher Scientific). Alternatively, slides that were stained with Alexa Fluor conjugated secondary antibody were counterstained and mounted with VECTASHIELD mounting medium for fluorescence with DAPI (Vector laboratory Inc, Burlingame, CA, USA) and were imaged under the microscope.

### Quantitative real-time RT/PCR

Performed as previously described [74] using total RNA isolated by TRIzol reagent (Invitrogen, Carlsbad, CA, USA) according to the manufacturer’s protocol. Briefly, 100ng of total RNA was analyzed by qRT/PCR carried out using iScript™ One-Step RT-PCR kit with SYBR Green (Bio-Rad, Hercules, CA, USA), according to the manufacturer’s instructions. Primer sequences are listed in Supplementary Table 3. Expression was normalized to *GAPDH* expression in each sample, and done in triplicates performed in 3 separate experiments.

### MSCs lineages staining assay

MSCs were seeded at a density of 1×10^5^ cells/well in a 6-well plate. MSCs were then grown in CM from HME, IRIS291, IRIS292, or IRIS293 cell lines for 7 days (media changed every day). After 7 days MSCs were washed with PBS and fixed in 10% formalin for 1h at RT. Cells were then washed thrice with PBS and stained for 1h for their differentiation into adipogenic using Oil-Red O (Sigma), osteogenic using Alizarin Red S (Sigma), chondrogenic using Alcian blue (Sigma) or fibrogenic using PicroSirius/Direct red 80 (Sigma) stain, according to the manufacturer’s instructions (Supplementary Table 4). Cells were washed with PBS and were photographed under light microscope. Experiments were done in triplicates performed 3 separate times.

### Endothelial tube formation assay

In matrigel-coated 96-wells, 5×10^4^ HUVEC cells were layered in EC medium supplemented with vehicle, 50ng/ml of recombinant human VEGF-A (rhVEGF-A, Sino Biological Inc., North Wales, PA, USA), or with 10μl of MSC-reconditioned or not IRIS291 or IRIS293 CM concentered 10 times using centricon (30K). Experiments were done in the presence or absence of 10nM of the VEGFR2 inhibitor “Ki8751” (10nM, TOCRIS Bioscience, Bristol, UK). After 6h of culture growth at 37°C images of tube formation were captured under light microscope. Experiments were done in triplicates performed 3 separate times.

### Hypoxyprobe staining

Mice were injected intraperitoneally with 60mg/kg pimonidazole solution (Hypoxyprobe™-1, Hypoxyprobe Inc., Burlington, MA, USA). One hour later, tumors were excised, paraffin embedded and cut into 5μm sections. Tissue sections were de-waxed, rehydrated and incubated in 3% H_2_O_2_ to quench endogenous peroxidase. After washing with PBS, antigen was retrieved by boiling in 10mM citrate buffer (pH 7.0) for 20min. Sections were cooled to RT, washed and blocked with 1% BSA for 1h at RT. Sections were incubated with anti-pimonidazole monoclonal antibody (MAb1), at a dilution of 1:50 in 1% BSA for 1h at RT. After washing, sections were incubated with the HRP-conjugated secondary antibody for 1h at RT. Reactivity was visualized using Vector DAB substrate kit (Vector laboratories Inc.). The sections were counterstained with Meyer’s hematoxylin for 2min, washed, dehydrated and mounted with Permount (Thermo Fisher Scientific). Sections were visualized under light microscope and photographed.

### Orthotopic mammary model

All animal experiments were approved by ‘Institutional Animal Care and Use Committee’ (IACUC) of University of Mississippi Medical Center and in accordance with the NIH guidelines. Nu/Nu (6-8 weeks old) female mice were injected with 1° IRISOE tumor cell line admixed or not with MSCs (at 10:1 ratio) in the mammary fat pad. Animals were monitored for tumor formation for 10 weeks. Tumors were measured every 3^rd^ day with digital caliper and tumor volume was measured according to the formula volume = (length × width^2^)/2. At the end of the experiment, mice were either sacrificed and tumor collected or underwent survival surgery to remove the tumors, and mice were monitored for metastasis formation using *in vivo* imaging (cells express luciferase). Excised tumors were paraffin embedded, sectioned and processed for IHC staining as described above.

### *In vivo* drug treatment

Twenty Nu/Nu mice were injected in mammary fat pads with 2×10^6^ IRIS293 tumor cells (derived from a 2° tumor generated from IRIS293 cells). After reaching a tumor volume of ∼250mm^3^, mice were divided into 4 different groups intraperitoneally injected with vehicle, IL-1R inhibitor “Anakinra” 10mg/kg/day (Swedish orphan biovitrum, SOBI, Stockholm, Sweden), CXCR2 inhibitor “SB265610” 2mg/kg/day (Tocris bioscience) or both at same concentrations for 4 consecutive days. On day 5, mice were sacrificed and serum and tumor were collected. Serum from naïve mice (non-tumor bearing) was also collected as a control for ELISA analyses. Tumors were digested into single cell suspension using collagenase-A and trypsin, and immediately frozen for later analysis. Animal studies were all done in a blinded fashion.

### Fluorescence-activated cell sorting

Single cell suspensions from the *in vivo* treated tumors were processed for FACS analysis. One million cells were stained with mouse specific anti-CD29 and anti-CD90 antibodies on ice for 1h. Cells were then washed thrice with FACS buffer (1% BSA in PBS) by centrifugation at 2000rpm at 4°C for 10min and further incubated with FITC-conjugated secondary antibody for 30min in ice. Cells were washed and then analyzed for surface staining on Gallios Flow Cytometer (Beckman Coulter, Pasadena, CA, USA) and data were analyzed using Kaluza Flow Cytometry Analysis Software v 1.2.

### TCGA, NIK and meta-analysis using kaplan Meier plotter

Meta-analysis based biomarker assessment using the online tool Kaplan Meier Plotter (http://kmplot.com/analysis) was used to delineate the association between gene expression of IL-1β, CCL2 and VEGF separately or combined with overall survival (OS), distant metastasis free survival (DMFS), and recurrent free survival (RFS) of breast cancer patient’s cohort. Within each cohort, high expresser and low expresser patients were analyzed and compared for their OS, DMSF and RFS, respectively.

### Statistical analysis

Statistical analysis was performed using unpaired, two-tailed Student’s t test. In all figures, data represents the mean from at least 3 separate biological repeats done in at least triplicates each +/- SD, ^*^P < 0.05, ^**^P < 0.01, and 8^***^P < 0.001.

## SUPPLEMENTARY MATERIALS FIGURES AND TABLES


