# Correction: A niche that triggers aggressiveness within BRCA1-IRIS overexpressing triple negative tumors is supported by reciprocal interactions with the microenvironment

**DOI:** 10.18632/oncotarget.23573

**Published:** 2017-12-22

**Authors:** Daniel Ryan, Abhilasha Sinha, Danielle Bogan, Joanna Davies, Jim Koziol, Wael M. ElShamy

**Affiliations:** ^1^ Breast Cancer Program, San Diego Biomedical Research Institute, San Diego, CA, USA; ^2^ University of Mississippi Medical Center, Jackson, MS, USA; ^3^ Department of Molecular and Experimental Medicine, Scripps Research Institute, San Diego, CA, USA

This article has been corrected: Dr. Sinha has been added in the author list with an equal contribution note along with Dr. Ryan and Dr. Bogan.

Original article: Oncotarget. 2017; 8:103182-103206. https://doi.org/10.18632/oncotarget.20892

