# Supplementary material for: A niche that triggers aggressiveness within BRCA1-IRIS overexpressing triple negative tumors is supported by reciprocal interactions with the microenvironment
Source: Oncotarget. 2017 Sep 14;8(61):103182–206. doi: 10.18632/oncotarget.20892 (PMC5732721; doi:10.18632/oncotarget.20892)
Supplement: Supplementary file 1 [file oncotarget-08-103182-s001.pdf]

A niche that triggers aggressiveness within BRCA1-IRIS overexpressing triple negative tumors is supported by reciprocal interactions with the microenvironment

SUPPLEMENTARY MATERIALS

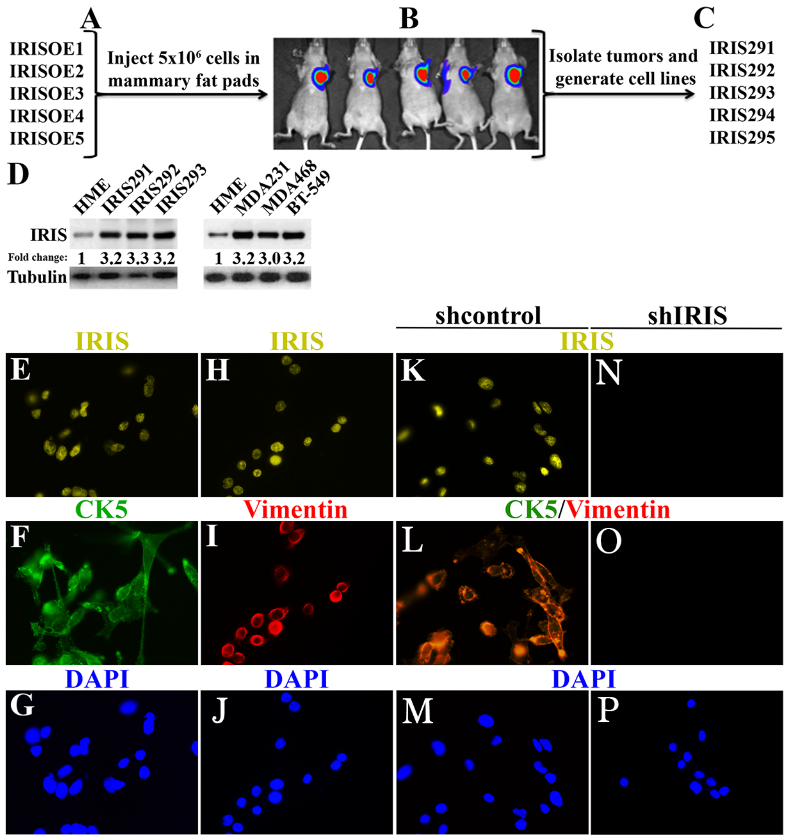

Supplementary Figure 1: Stepwise generation of 1º orthotopic IRISOE mammary tumor cell lines. Briefly, naïve HME cells Dox-inducibly overexpressing IRIS (5 clones IRISOE1-IRISOE5) were injected in SCID mice mammary fat pads **(A)**. Three month later tumors developed **(B)** were resected and propagated in culture (**(C)**, named IRIS291-IRIS295). **(D)** Expression of Dox-inducible IRIS in IRIS291, IRIS292, and IRIS293 (left) as compared to 3 TNBC cell lines (MDA-MB-231, MDA-MB-468, and BT-549). Note both are compared to IRIS level in naïve HME cells. Fluorescent staining of tumor cells dissociated from 1º IRISOE orthotopic mammary tumors (in the presence of Dox), with mouse anti-IRIS, guinea pig anti-CK5, and rabbit anti-vimentin. Goat anti-mouse Alexa Fluor 532, anti-guinea pig Alexa Fluor 488, and anti-rabbit Alexa Fluor 568 were used as 2º antibodies. Note expression of CK5 in every IRISOE tumor cells **(E-G)**, vimentin in every IRISOE tumor cell **(1H-1J)**, co-localization of CK5 and vimentin in every IRISOE tumor cell **(1K-1M)**, and lack thereof in IRIS silenced cells "1N-P".

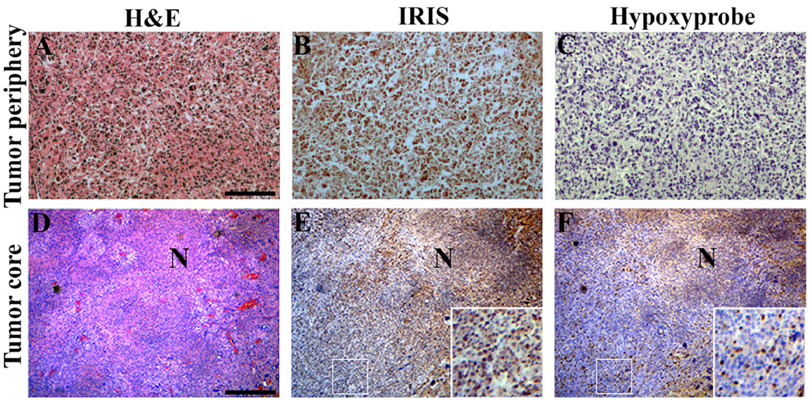

Supplementary Figure 2: Staining of IRISOE 1º orthotopic mammary tumor section of tumors' periphery or core showing prevalence of necrotic/hypoxic core (aggressiveness niche). (**A** and **D**) H&E, (**B** and **E**) staining mouse anti-IRIS antibody, and (**C** and **F**) hypoxyprobe staining. N: denotes the necrotic core within the tumor. Scale bars: 100µm in A-C, and 500µm in D-F.

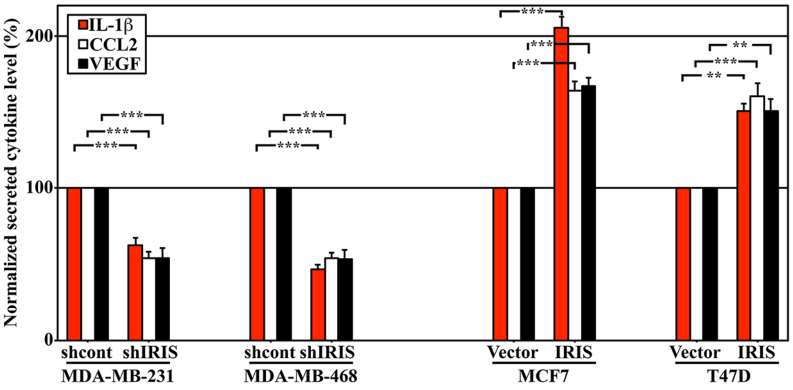

Supplementary Figure 3: Levels of IL-1β (red bars), CCL2 (white bars), and VEGF (black bars) secreted from the TNBC tumor cell lines; MDA-MB-231 and MDA-MB-468 expressing shcontrol or shIRIS, as well as from the luminal A/ERα+ breast cancer cell lines: MCF7, T47D expressing vector or IRIS.

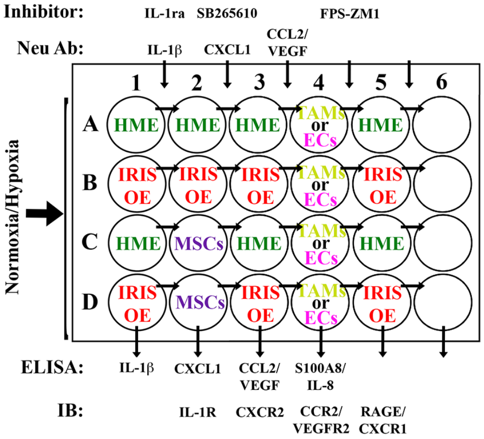

Supplementary Figure 4: Schematic representation of the *in vitro* experiments performed in this study.

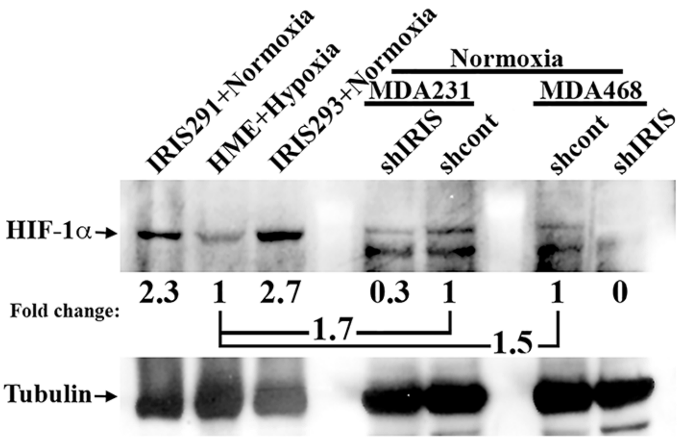

Supplementary Figure 5: **Expression of HIF-1α in hypoxic naïve HME and normoxic IRIS291 or IRIS293, as well as normoxic MDA-MB-231, or MDA-MB-468 cells expressing control or IRIS shRNA.**

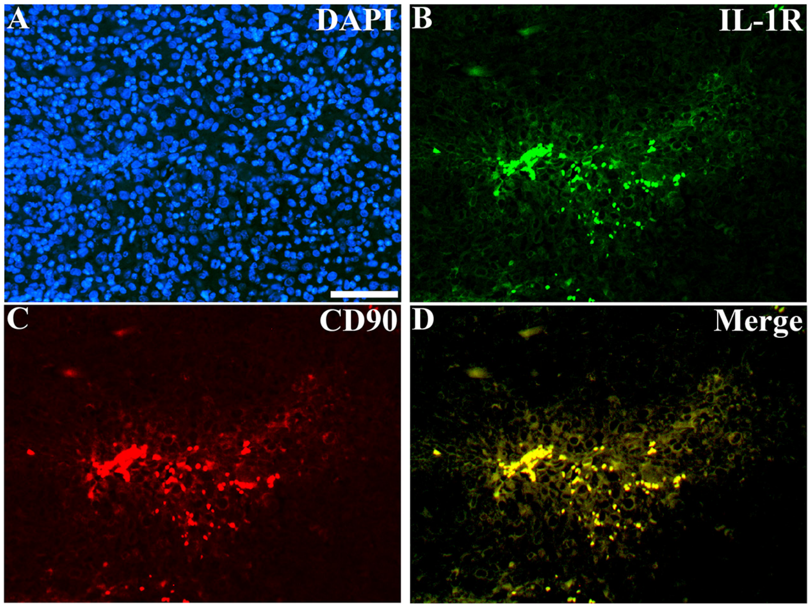

Supplementary Figure 6: Staining of IRIS293 tumor sections with DAPI **(A),** IL-1R **(B)** and the MSC marker CD90 **(C). (D)** Shows merged images. Scale bars: 50μm in A-D.

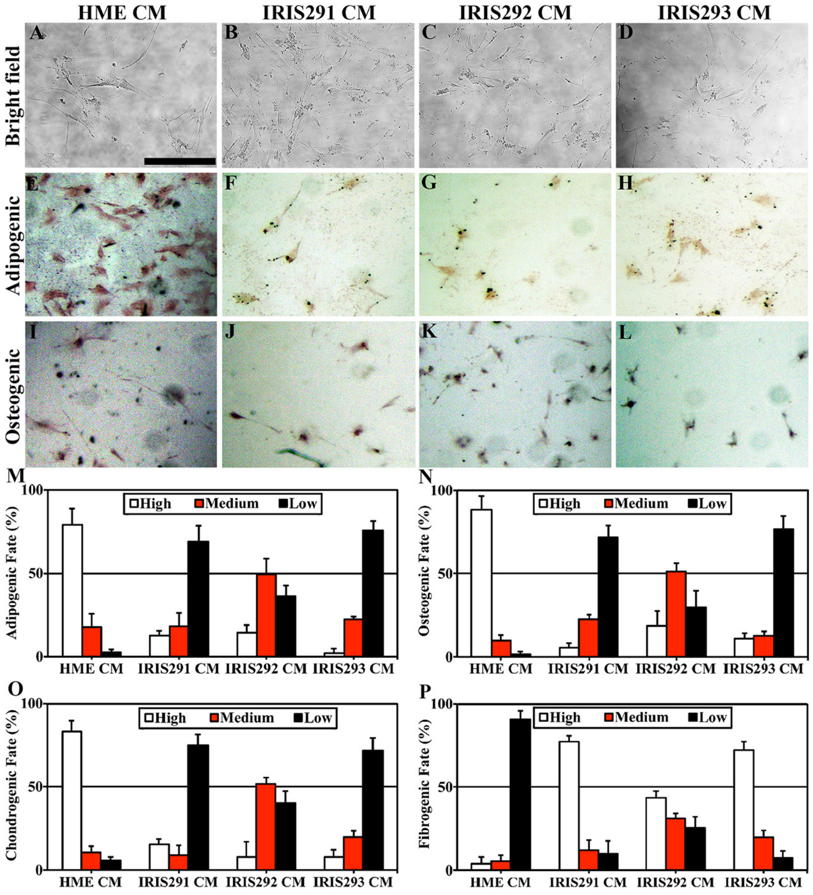

Supplementary Figure 7: Multi-lineage differentiation staining of MSCs. **(A-D)** Bright field microscopy, **(E-H)** Oil Red O adipogenic staining, and **(I-L)** Alizarin osteogenic staining for MSCs grown for 7 days in CM from naïve HME, IRIS291, IRIS292 or IRIS293 cells. **(M-P)** Percentage of MSCs showing high, medium, or low intensity staining for the different lineages dye following exposure to indicated CM for 7 days. Scale bars, 10µm in A-L.

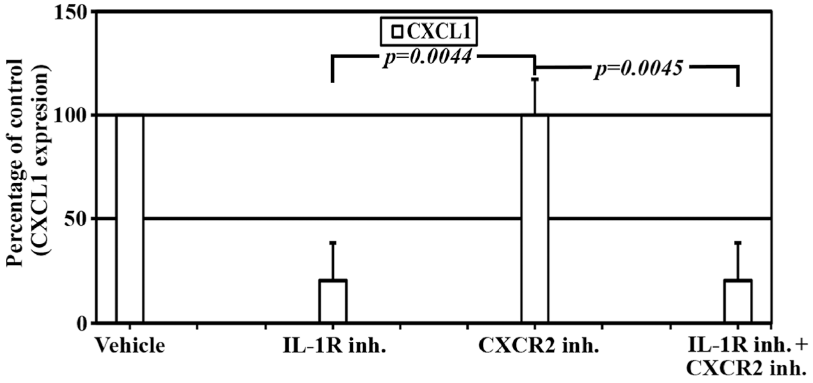

Supplementary Figure 8: Percentage CXCL1 protein expression in vehicle, IL-1R inhibitor, CXCR2 inhibitor, or both drugs treated tumors.

Supplementary Table 1: List of antibodies used in the study and their sources.

Antibody	Species	Catalog no.	Company
IRIS	Mouse	-	Developed in our lab
AKT (S^{473})	Rabbit	4060	Cell Signaling Inc. (Danvers, MA, USA)
EGFR	Rabbit	9922	Cell Signaling Inc.
p-ERK (T^{202}/Y^{204})	Rabbit	4370	Cell Signaling Inc.
p-p38 (T^{180}/Y^{182})	Rabbit	9211	Cell Signaling Inc.
Slug (C19G7)	Rabbit	9585	Cell Signaling Inc.
Sox2 (D6D9)	Rabbit	3579	Cell Signaling Inc.
CK17	Rabbit	ab51056	Abcam Inc. (Cambridge, MA, USA)
γ-Tubulin	Rabbit	ab11321	Abcam Inc.
MRP8 (S100A8)	Rabbit	ab92331	Abcam Inc.
RAGE (EPR12206)	Rabbit	ab172473	Abcam Inc.
VEGFR2	Rabbit	ab2349	Abcam Inc.
CCR2	Rabbit	ab155321	Abcam Inc.
IL-1RA	Rabbit	ab2573	Abcam Inc.
Integrin beta 1/CD29	Rat	ab95623	Abcam Inc.
CD31	Rat	ab56299	Abcam Inc.
CD90/Thy1	Rat	ab3105	Abcam Inc.
F4/80 (CI:A3-1)	Rat	ab6640	Abcam Inc.
CXCR1	Rabbit	ab137351	Abcam Inc.
CXCR2	Mouse	ab24963	Abcam Inc.
CK5/6 (D5/16 B4)	Mouse	ab17133	Abcam Inc.
CK5	Guinea pig	ab1941135	Abcam Inc.
Twist (Twist2C1a)	Mouse	ab50887	Abcam Inc.
VEGF (VG-1)	Mouse	ab1316	Abcam Inc.
CXCL1	Rabbit	bs-0863R	Bioss Inc (Woburn, MA, USA)
CCL2	Mouse	MAB679	R&D Systems, Minneapolis, MN, USA
Cortactin	Mouse	610049	BD Biosciences, (San Jose, CA, USA)
N-cadherin	Mouse	610920	BD Biosciences
IL-8	Mouse	554717	BD Biosciences
IL-1β	Rabbit	P420B	Thermo Fisher Scientific (Waltham, MA, USA)
NF-κB/p65	Mouse	IMG-150A	Imgenex (San Diego, CA, USA)
Actin	Mouse	CP01	Calbiochem (San Diego, CA, USA)
Vimentin/LN6	Mouse	(Ab-1, IF01)	Calbiochem
Vimentin	Rabbit	Ab45939	Abcam Inc.

Supplementary Table 2: List of siRNAs used in the study and their sources.

siRNA	Catalog no.	Company
HIF-1α siRNA (h)	AM16708	Thermo Fisher Scientific (Waltham, MA, USA)
Silencer® Firefly Luciferase (GL2 + GL3) siRNA	AM4629	

Supplementary Table 3: Sequences of primers used in qRT/PCR in the current studies.

Gene	Primers sequence
CXCL1	Forward: 5′-AACCGAAGTCATAGCCACAC-3′
	Reverse: 5′-GTTGGATTTGTCACTGTTCAGC-3′
IL-12	Forward: 5′-AAAGGACATCTGCGAGGAAAGTTC-3′
	Reverse: 5′-CGAGGTGAGGTGCGTTTATGC-3′
CXCL-10	Forward: 5′-GAAAGCAGTTAGCAAGGAAAGGTC-3′
	Reverse: 5′-ATGTAGGGAAGTGATGGGAGAGG-3′
CCL-17	Forward: 5′-CGGGACTACCTGGGACCTC-3′
	Reverse: 5′-CCTCACTGTGGCTCTTCTTCG-3′
CCL-18	Forward: 5′-ATGGCCCTCTGCTCCTGT-3′
	Reverse: 5′-AATCTGCCAGGAGGTATAGACG-3′
GAPDH	Forward: 5′-AATGGAAATCCCATCACCATCT-3′
	Reverse: 5′-CGCCCCACTTGATTTTGG-3′

Supplementary Table 4: Staining protocol for MSC differentiation lineage for MSCs grown for 7 days in CM from HME or IRISOE cells.

Lineage type	Staining protocol
Adipogenic lineage	Oil-Red O (Sigma)
Osteogenic lineage	Alizarin Red S (Sigma)
Chondrogenic lineage	Alcian blue (Sigma)
Fibrogenic lineage	PicroSirius/Direct red 80 (Sigma)
